# Polydopamine Applications in Biomedicine and Environmental Science

**DOI:** 10.3390/ma17163916

**Published:** 2024-08-07

**Authors:** Hossein Omidian, Renae L. Wilson

**Affiliations:** Barry and Judy Silverman College of Pharmacy, Nova Southeastern University, Fort Lauderdale, FL 33328, USA; rw1273@mynsu.nova.edu

**Keywords:** polydopamine, tissue engineering, cancer treatment, pollutant adsorption, biocompatibility

## Abstract

This manuscript explores the multifaceted applications of polydopamine (PDA) across various scientific and industrial domains. It covers the chemical aspects of PDA and its potential in bone tissue engineering, implant enhancements, cancer treatment, and nanotechnology. The manuscript investigates PDA’s roles in tissue engineering, cell culture technologies, surface modifications, drug delivery systems, and sensing techniques. Additionally, it highlights PDA’s contributions to microfabrication, nanoengineering, and environmental applications. Through detailed testing and assessment, the study identifies limitations in PDA-related research, such as synthesis complexity, incomplete mechanistic understanding, and biocompatibility variability. It also proposes future research directions aimed at improving synthesis techniques, expanding biomedical applications, and enhancing sensing technologies to optimize PDA’s efficacy and scalability.

## 1. Introduction

### 1.1. Polydopamine Structure and Synthesis

**Figure 1:**
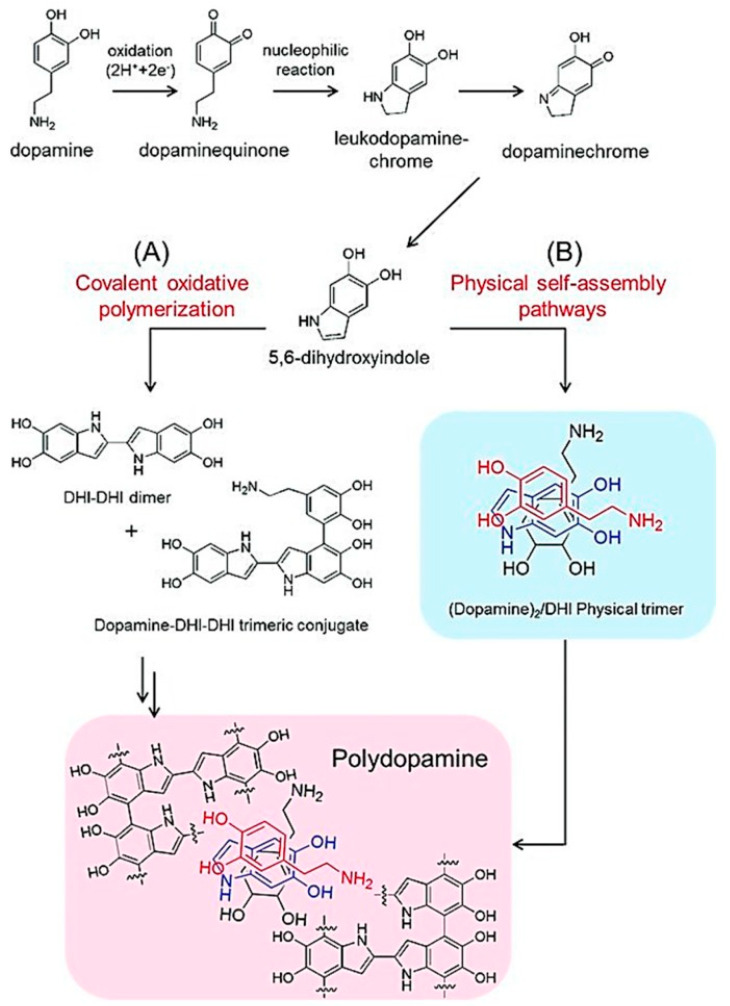
A schematic representation hypothesized as the formation PDA. (**A**) Formation of covalent bonds via oxidative polymerization (**B**) Physical self-assembly reaction of intermediates and dopamine. Adopted with permission [7].

The synthesis of polydopamine is inspired by the natural process of melanin formation in mussels, which utilize dopamine to create strong, adhesive coatings [1,2,3,4]. The pathway for synthesizing polydopamine can be broadly categorized into two main mechanisms: covalent oxidative polymerization and physical self-assembly pathways. In the covalent oxidative polymerization pathway, dopamine undergoes an initial oxidation step to form dopaminequinone [5,6,7]. This intermediate then undergoes a series of nucleophilic reactions to produce leucodopaminechrome and subsequently dopaminechrome [7,8]. The further oxidation and intramolecular cyclization of dopaminechrome lead to the formation of 5,6-dihydroxyindole (DHI). DHI molecules can then polymerize through covalent bonds to form DHI-DHI dimers and dopamine-DHI-DHI trimeric conjugates, ultimately leading to the formation of polydopamine [9]. The physical self-assembly pathway involves non-covalent interactions among dopamine and its oxidized derivatives. During this process, dopamine and DHI can interact through hydrogen bonding, π-π stacking, and other non-covalent interactions to form supramolecular assemblies. These assemblies further aggregate to form polydopamine structures without the need for covalent bonding (Figure 1) [7].

The uncontrolled self-polymerization of PDA can lead to severe interparticle aggregation, which impacts both application performance and the environment. Controlled synthesis methods, such as using sodium borohydride (NaBH4) as a reductant, effectively control PDA polymerization. This method was shown to form a thin PDA coating on mixed cellulose ester (MCE) membranes, improving water transport performance and organic foulant removal while exhibiting excellent chemical stability and antimicrobial activity. This approach reduces environmental impact by optimizing resource use and minimizing waste. The controlled synthesis of PDA using NaBH4 demonstrated significant potential in water purification, achieving approximately 90% bovine serum albumin rejection, thus contributing to environmental protection by enhancing water purification systems [10].

PDA’s properties enable the development of functional metal-containing PDA (MPDA) nanomaterials used in various applications, like catalysis and medical imaging. The proper synthesis and coordination of MPDA can reduce waste and energy consumption in industrial processes, lowering their environmental footprint. Research has focused on improving synthesis processes to enhance functionality and reduce environmental impact. Future directions include exploring new reductants and oxidants, developing greener synthesis methods, and expanding the applications of PDA-based materials in environmental protection [11].

By adopting controlled synthesis strategies and environmentally beneficial applications can significantly reduce the potential negative impact of polydopamine. These considerations are integrated into our manuscript.

### 1.2. General Polydopamine Properties and Applications

Polydopamine (PDA) has emerged as a significant material in biomedical engineering and materials science due to its unique properties and versatility, addressing several critical gaps and challenges. A primary motivation for utilizing PDA in biomedical applications is its exceptional biocompatibility and ability to enhance the bioactivity of scaffolds and implants. Researchers have focused on the development of PDA coatings to improve the integration and functionality of medical devices within biological systems. These coatings facilitate cell adhesion, differentiation, and proliferation, which are crucial for successful tissue engineering and regenerative medicine [1,12,13,14,15,16,17,18,19,20]. Additionally, the antibacterial properties of PDA help reduce the risk of infections associated with medical implants, addressing a significant challenge in post-surgical complications [21,22,23,24,25].

In biosensing and diagnostics, PDA has been employed to overcome the limitations of conventional sensing platforms, which often involve complex, multi-step procedures or lack sensitivity and specificity. The introduction of PDA has enabled the development of more stable, effective, and user-friendly sensors for critical applications such as early disease detection, environmental monitoring, and safety. These PDA-based sensors demonstrate enhanced detection capabilities for a variety of biological and chemical targets [2,26,27,28,29,30,31,32,33,34,35].

Material engineering also benefits from the incorporation of PDA due to its excellent adhesive properties and ease of functionalization. Researchers have leveraged PDA to enhance the mechanical, tribological, and surface properties of various materials, leading to improvements in durability and performance in environmental and industrial applications. This includes the development of surfaces that are superhydrophobic, antimicrobial, and antifouling, which are essential for extending the lifespan and functionality of materials exposed to harsh conditions [3,4,36,37,38,39,40,41,42].

In nanotechnology and advanced material synthesis, PDA is frequently used to modify the surface properties of nanoparticles, facilitating their application in catalysis, drug delivery, and environmental remediation. The ability of PDA to form stable coatings on nanoparticles enhances their biocompatibility and functional performance, making them suitable for a wide range of applications [9,31,42,43,44,45,46,47,48,49,50]. For instance, PDA-coated gold nanoparticles (GNP@PDAs) exhibit low cytotoxicity, maintain structural integrity within cells, and demonstrate long-term stability in vivo, without notable histological toxicity to major organs, such as the liver and spleen [51]. This stability and biocompatibility are crucial for ensuring the safe and effective delivery of therapeutic agents.

A notable advancement in this field includes the synthesis of antibacterial carbon quantum dots (CQDs) through a one-step pyrolysis of biogenic polyamine (PA) and dopamine (DA) mixture. SPM/DA-CQDs synthesized from DA combined with spermine (SPM) exhibit effective antibacterial activity and high adhesion properties on glass and surfaces of polymeric contact lens material. The antimicrobial activity of SPM/DA-CQDs is primarily due to their ability to disrupt the bacterial membrane, and they possess wide spectrum antimicrobial activity against both Gram-negative and Gram-positive bacteria. The great biocompatibility and antibiofilm properties of SPM/DA-CQDs suggest their potential as coating materials to protect medical devices from contamination [52].

Further studies have shown that PDA coatings significantly reduce adverse biological responses. PDA-modified surfaces, such as those on poly-L-lactic acid and quantum dots, diminish inflammatory and immunological reactions, enhancing the overall safety profile of these materials when in contact with tissues or blood [53]. PDA’s inherent properties, including high photothermal conversion efficiency, versatile adhesion, and excellent drug binding capacity, make it a highly suitable material for advanced drug delivery systems and therapeutic applications, such as photothermal therapy, bioimaging, and biosensing [54,55].

However, the potential neurotoxicity of PDA nanoparticles (NPs) has also been a subject of investigation. Studies using zebrafish larvae have indicated that, while PDA NPs are generally stable and biocompatible, high concentrations can lead to neurotoxic effects, such as inhibited axonal growth and impaired motor function. Despite these findings, no apoptosis of central neurocytes was observed, suggesting that PDA’s neurotoxic effects are dose-dependent and primarily affect peripheral motor neurons [56]. These insights underline the importance of thorough toxicity assessments to ensure the safe biomedical application of PDA-based nanomaterials.

PDA’s chemical attributes facilitate a broad spectrum of applications. These include surface modification, the enhancement in bioactivity, the development of composite materials and functional nanocomposites, and the immobilization of biomolecules for sophisticated biosensing technologies. Its impact is profound across both the environmental [9,45] and biomedical [9,57,58] fields, where an enhanced material performance can lead to significant advancements in areas like pollution control, drug delivery, and tissue engineering. Extensive research into PDA’s biocompatibility and safety underlines its potential as a versatile and effective material for a wide range of biomedical applications, from targeted drug delivery and cancer therapy to biosensing and imaging, provided that appropriate dosing and safety measures are maintained [59].

The selection of PDA modification methodologies is a complex process influenced by a multitude of factors tailored to the specific requirements of the intended application. Whether for biomedical, environmental, or industrial purposes, considering aspects such as biocompatibility, mechanical properties, environmental impact, and practical feasibility ensures that PDA-based materials perform optimally in their respective contexts. By carefully aligning these methodologies with the unique needs of each application, researchers can enhance the efficacy and functionality of PDA-modified materials, achieving desired outcomes across various fields. Recent studies further illustrate the broad utility of PDA and its analogues in diverse applications. For instance, the extensive use of implants in orthopedic surgeries has driven a tremendous demand for surface modifications to enhance implant function and reduce surgery failure. PDA, with its ability to adhere to various substrates and immobilize biomolecules and metal ions, has proven beneficial in modulating cellular responses, such as spreading, migration, proliferation, and differentiation. This enhances the function and osseointegration of orthopedic implants while providing antimicrobial properties [60]. Polydopamine analogues (PDANAs) expand the capabilities of PDA by incorporating additional functional groups, achieved through post-functionalization, oxidative polymerization, or copolymerization with other monomers or reactive polymers. This broadens the range of properties and applications for these materials, highlighting their future potential in various fields [61]. In membrane technology, functional groups on support membranes are crucial for high-performance membrane adsorbers (Mas). Surface modifications, such as alkali treatment, oxidation, and PDA deposition, improve adsorption performance. For instance, PDA deposition on membranes enhances protein adsorption capacity, offering potential advancements in this area [62]. PDA hydrogels also demonstrate significant promise, with their abundant functional groups allowing for various covalent and non-covalent interactions with polymers and transition metal ions. These interactions impart diverse functions to hydrogels, such as adhesion, photothermal effects, ultraviolet protection, antioxidant ability, and antibacterial properties, enabling their application across the biomedical, environmental, energy, and electronic fields [63].

### 1.3. Biocompatibility, Biosafety, and Long-Term Effects of Polydopamine for Biomedical Applications

In-Vivo and In-Vitro Evaluation: Polydopamine (PDA) coatings have shown significant promise in surface functionalization for biomedical applications due to their unique properties. PDA forms a nanometer-scale organic thin film on various material surfaces, facilitating the attachment of proteins, peptides, oligonucleotides, metal ions, or synthetic polymers. This makes it particularly suitable for nanomedicine. Evaluating its toxicity is crucial. Studies have demonstrated that PDA acts as a biocompatible layer, reducing inflammatory and immunological responses in vivo, indicating its potential to mitigate the adverse biological effects of coated materials [53].

Reactive Oxygen Species (ROS) Scavenging: PDA nanoparticles have emerged as effective ROS scavengers, which is critical in cellular metabolism and oxidative stress-related diseases. Among different types of PDA nanoparticles (solid, mesoporous, and hollow), mesoporous PDA exhibited the highest ROS scavenging capability due to its specific surface area. In vivo studies on inflammation models confirmed that mesoporous PDA effectively reduced reperfusion injury, improved renal function, and prevented periodontitis progression, highlighting its biosafety and biocompatibility [64].

Biocompatibility and Drug Delivery: Polydopamine has been utilized to enhance the biocompatibility and efficacy of drug delivery systems. For instance, PDA-coated Zein nanoparticles for gambogenic acid delivery significantly improved water solubility, reduced vascular irritation, and enhanced tumor targeting and bioavailability in vivo, demonstrating good cytocompatibility and reduced irritation [65]. Additionally, PDA-coated cobalt ferrite nanoparticles have shown potential for drug delivery, exhibiting low toxicity in vitro and effective tumor inhibition in vivo [66].

Vascular Applications: For cardiovascular applications, modified acellular blood vessels with bivalirudin via PDA coating showed excellent cytocompatibility, blood compatibility, and reduced inflammation in vivo, suggesting potential as clinical substitutes for small-diameter vascular grafts [67].

Osteogenic Induction: PDA-assisted hydroxyapatite coating on polyamide 66 substrates improved osteogenic induction both in vitro and in vivo, enhancing cell adhesion, proliferation, and bone formation around implants [68].

Toxicity Studies: In the context of photothermal therapy (PTT), mesoporous PDA (MPDA) was evaluated for safety in clinical applications. MPDA exhibited excellent biocompatibility with no significant impact on intestinal microflora in vivo, supporting its potential for clinical use [63]. Subacute toxicity studies of MPDA indicated that low and medium doses were safe, while high doses led to disturbances in the gut microbiota and inflammation, emphasizing the need for dose control in clinical applications [69]. Furthermore, PDA’s biocompatibility has been affirmed in various studies, showing minimal cytotoxic effects and significant potential for safe biomedical applications [53,70].

Kinetic Studies: The synthesis and characterization of polydopamine using dopamine monomer and (hydroxymethyl)aminomethane (TRIS) as the oxidant have been studied to understand the kinetics of dopamine polymerization. The effect of the temperature and TRIS concentration on the kinetics, along with the morphological, structural, and thermal properties of polydopamine, were evaluated, providing insights into optimizing the synthesis process for various applications. In this study, Fickian diffusion was observed for the investigated imprinted polymer of polydopamine/graphene oxide as a drug carrier for rivastigmine [71]. This diffusion model was also found for polydopamine-coated cobalt ferrite nanoparticles (PDA-coated CF-NPs) [66].

## 2. Coatings and Surface Modifications

**Figure 2:**
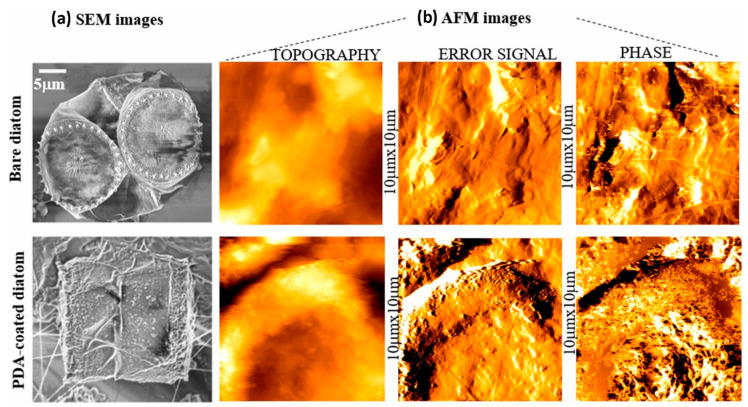
(**a**) SEM images and (**b**) AFM images of PDA-coated diatom and bare diatom cells at the scale of 10 μm × 10 μm. A bioinspired biosensing polydopamine layer was investigated for the functionalization of silicon-based photonic biosensors. This approach aims to enhance the efficiency of biomolecule immobilization on the sensors, thereby boosting their performance in detecting low concentrations of biomolecules in human serum. The studies indicate that PDA coatings offer high compatibility and resistance to hydrolysis, ensuring consistent biofunctionalization across a range of biomarkers. This development is pivotal for advancing the sensitivity and reliability of biosensors, crucial in medical diagnostics. Reprinted (adapted) with permission from Hasani-Sadrabadi, et al. (2019). Copyright 2019 American Chemical Society [73].

The exploration of polydopamine (PDA) coatings in various applications, especially in environmental protection and enhancement in mechanical properties across diverse substrates, has led to several innovations. One intriguing application is the coating of living diatom microalgae with PDA, which not only enhances their resistance to environmental stresses but also minimizes their uptake of staining agents [72]. This modification has promising implications for the development of biosensors and devices that utilize living cells, providing enhanced durability in harsh environments where traditional sensors might fail. The improved resilience of these coated diatoms opens new avenues for environmental monitoring and biotechnological applications (Figure 2) [73].

Further research into the effects of PDA on surface mineralization has uncovered its significant role in influencing the nucleation modes of calcium phosphate on polymeric substrates. PDA coatings have been found to enhance the early stages of nucleation, thus improving the bond strength at the mineral–substrate interface. These findings suggest that PDA could play a vital role in boosting the interfacial strength of biomaterials, essential for applications in biomedical implants and related technologies [74].

In biomedical applications, the ongoing innovation in surface modifications using PDA has led to significant advancements, particularly in enhancing the hydrophobicity and antimicrobial properties of medical devices. A notable development involves the immobilization of highly hydrophobic polytetrafluoroethylene particles on surfaces using PDA and nitric oxide (NO)-releasing polymers. This multifunctional surface is designed to reduce bacterial infections and platelet adhesion on biomedical devices. The cobblestone-like structure of the surface has been shown to reduce adhered bacteria by 99% and platelet adhesion by 83% in vitro, demonstrating its potential to significantly improve the safety and efficacy of medical devices [75].

Additional research has facilitated the creation of antifouling and antimicrobial polymer membranes by coating polypropylene with PDA and subsequently functionalizing it with poly(N-vinyl pyrrolidone) (PVP). After iodine complexation with PVP, these membranes exhibit enhanced hydrophilicity, wettability, and antimicrobial activity. Such improvements render the membranes highly effective in resisting bacterial growth and fouling, extending their utility in various medical and industrial applications [76].

The enhancement in antibacterial performance on Foley catheters has also been achieved through PDA surfaces conjugated with amino acids such as lysine and alpha-aminoisobutyric acid. This functionalization significantly reduces bacterial attachment and growth on the surfaces of both polyethylene terephthalate (PET) films and Foley catheters. The modified surfaces demonstrate a notable increase in antibacterial activity against both Gram-positive and Gram-negative bacteria, providing a reliable strategy to mitigate infections associated with catheter use [22].

Furthermore, antibacterial composites have been developed using PDA to deposit silver nanoparticles on calcium phosphate substrates. These composites, namely octacalcium phosphate decorated with silver (OCPdAg) and alpha-tricalcium phosphate decorated with silver (alphaTCPdAg), show improved biocompatibility and antibacterial effectiveness, supporting osteoblast activity while inhibiting bacterial growth without being toxic to the osteoblasts. This suggests a promising route for developing bone implants with enhanced infection resistance [25].

In addition, the creation of a universal antimicrobial and antifouling coating combines PDA with antimicrobial copper ions and zwitterionic sulfobetaine. This bioinspired coating enhances both antimicrobial and antifouling properties on various substrates, such as SiO_2_, TiO_2_, gold, plastics, and Nitinol alloy, proving effective in preventing bacterial colonization on medical devices. The coating has shown a significant reduction in bacterial adsorption and living bacteria on coated catheters, offering a versatile and effective solution for reducing infection risks associated with a wide range of medical devices [24].

In terms of mechanical properties and for environmental applications, PDA nanocoatings have benefited significantly from thermal annealing. This process enhances the intermolecular and cohesive interactions within the coatings, leading to improved scratch resistance while preserving the functionality necessary for secondary coating reactions. Such mechanically robust coatings are crucial for applications requiring durable protective layers, such as in the automotive or aerospace industries [36].

The development of stable superhydrophobic coatings on wood surfaces exemplifies the versatility of PDA when combined with electroless deposition techniques. These coatings create a hierarchical roughness structure on the wood, resulting in exceptional durability and maintaining superhydrophobic properties even under severe conditions. The demonstrated stability under tests such as ultraviolet aging, immersion, and boiling confirms their effectiveness and potential for protecting wood in outdoor and marine environments. The achieved water contact angle of 157° highlights the efficacy of these coatings in repelling water, which is essential for maintaining the integrity and longevity of wood structures [39].

To summarize, the extensive research on polydopamine (PDA) coatings and surface modifications highlights its versatile applications across various fields. A shared focus among studies is the enhancement in mechanical and protective attributes, such as improved resilience to environmental stress in diatom microalgae, which is crucial for biosensor applications [72]. Similarly, PDA’s role in biomolecule immobilization on silicon-based photonic biosensors enhances detection sensitivity, vital for medical diagnostics [73]. In biomedical contexts, PDA facilitates the early nucleation of calcium phosphate on polymers, boosting interfacial strength for implants [74], and significantly reduces bacterial and platelet adhesion when combined with hydrophobic polymers, addressing infection risks in medical devices [75]. Differences arise in the specific applications and combined materials, such as PDA-coated polypropylene membranes functionalized with PVP for enhanced antimicrobial activity [76], and Foley catheters conjugated with amino acids to reduce bacterial attachment [22]. Moreover, antibacterial composites with PDA-deposited silver nanoparticles improve biocompatibility for bone implants [25], while universal antimicrobial PDA coatings with copper ions and sulfobetaine demonstrate broad effectiveness against bacterial colonization [24]. Additionally, the thermal annealing of PDA nanocoatings enhances mechanical robustness for automotive and aerospace uses [36], and superhydrophobic PDA coatings protect wood surfaces in harsh environments, showing promise for outdoor and marine applications [39].

Table 1 summarizes concerns and limitations associated with the application of polydopamine coatings in various fields, including potential impacts on ecological functions, long-term stability, durability, effectiveness, cytotoxicity, environmental impact, thermal stability, and consistency in functionalization.

## 3. Bone Tissue Engineering

Bone tissue engineering is a dynamically evolving discipline that ties materials science and cellular biology, aimed at enhancing bone repair and regeneration. This field has seen significant advancements through the integration of innovative materials and biological techniques.

One advancement is the surface modification of calcium silicate with polydopamine (PDA). The functionalization with PDA has markedly improved the performance of Wharton’s jelly mesenchymal stem cells (WJMSCs) by boosting cell adhesion, enhancing the secretion of the extracellular matrix, and up-regulating the expression of genes and proteins pivotal for osteogenesis [12]. These cellular function enhancements correlate directly with the PDA concentration used in the modification process, highlighting the material’s crucial role in bone tissue engineering.

Further explorations into polydopamine’s applications have extended to the development of biomimetic materials. For instance, when integrated into a hydroxyapatite/collagen (HC) composite, PDA has been shown to augment the mechanical strength of the material while simultaneously promoting cell proliferation and mineralization [13]. These modifications not only yield superior in vitro outcomes—including enhanced cell attachment and proliferation—but also significantly foster bone regeneration in vivo, especially around particulates.

**Figure 3:**
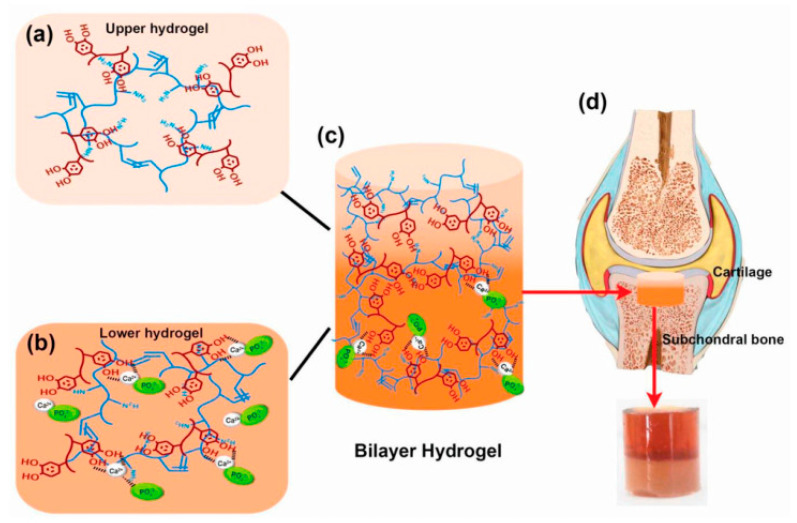
A schematic illustration of (**a**) gelatin methacryloyl (GelMA) with polydopamine (PDA) upper hydrogel layer for cartilage defect repair and (**b**) Ca^2+^-GelMA with PO_4_^3−^-GelMA to generate a hydroxyapatite (HA) layer hydrogel for subchondral bone repair. (**c**) The combination of both hydrogels to form the final structure of the hydrogel bilayer. (**d**) The application of the hydrogel bilayer for bone defect repair. Adopted with permission [1].

Polydopamine also plays a crucial role in the biomineralization process of hydroxyapatite on various implant materials. A comprehensive review has underlined PDA’s efficacy in enhancing biocompatibility, mechanical strength, and corrosion resistance of these materials, which are fundamental for the repair and regeneration of bone defects (Figure 3) [1].

Moreover, the osteointegration of titanium alloy (Ti6Al4V) scaffolds can be considerably enhanced through a PDA-assisted hydroxyapatite coating. This specialized coating on porous Ti6Al4V scaffolds has demonstrated significant improvements in cell attachment and proliferation, as well as bone regeneration in vivo [14]. The scaffolds coated with this material show increased expression of osteogenic markers, offering superior osteointegration compared to their uncoated counterparts.

The utility of polydopamine extends into more intricate biomimetic structures. Utilizing a honeycomb-like template derived from mollusk shells, researchers have created hydroxyapatite microspheres within a PDA matrix. This HA/pDA composite not only promotes mineral nucleation but also supports osteoblast activity, thereby enhancing gene expression vital for subchondral bone regeneration [18]. The mechanical strength and osteogenic activity of this composite make it highly suitable for applications in subchondral bone repair.

In another approach, the immobilization of BMP-2 peptides on scaffolds via polydopamine coating has led to the development of polydopamine-coated PLGA-[Asp-PEG]n scaffolds. These scaffolds sustain the delivery of BMP-2-derived peptides, significantly enhancing osteogenic differentiation and promoting ectopic bone formation. This method has proven more effective in osteogenic differentiation of rat mesenchymal stem cells (rMSCs) and has shown to be effective in enhancing bone formation due to the sustained release of these peptides [15].

Moreover, the creation of polydopamine-coated biomimetic bone scaffolds loaded with exosomes represents a promising strategy in bone regeneration. Scaffolds of the SF-CS-nHA type have been successfully used to boost bone marrow stem cell (BMSC) proliferation and osteogenic differentiation, significantly improving bone regeneration in rabbit models [19]. These scaffolds have demonstrated notable improvements in osteogenic effects and bone defect repair compared to control groups.

To conclude, research on the integration of polydopamine (PDA) into bone tissue engineering materials consistently shows enhanced cell adhesion, proliferation, and osteogenic differentiation. PDA-modified calcium silicate and hydroxyapatite/collagen composites improve mechanical strength and promote extracellular matrix secretion and gene expression crucial for bone regeneration [12,13]. Similarly, PDA-assisted hydroxyapatite coatings on titanium alloys exhibit superior osteointegration and in vivo bone regeneration [14], highlighting PDA’s role in enhancing the biocompatibility and mechanical properties of bone scaffolds. Differences among studies often relate to the degree of osteogenic improvements, likely influenced by variations in PDA concentration and material compositions. For instance, while some research underscores PDA’s effectiveness in creating biomimetic structures and immobilizing BMP-2 peptides, the outcomes vary, suggesting a need for standardized protocols to optimize results [15,19]. Despite these advancements, significant gaps remain, particularly in understanding the long-term in vivo performance and the scalability of these biomaterials for clinical applications. This indicates the necessity for more comprehensive longitudinal studies to fully achieve the clinical potential of PDA-enhanced bone scaffolds.

Table 2 summarizes the concerns and limitations associated with the modification of biomaterials using polydopamine (PDA), highlighting areas of uncertainty regarding its long-term effects, stability, inflammatory potential, degradation behavior, wear debris, peptide release kinetics, variability in exosomes, and scaffold biocompatibility and mechanical stability.

## 4. Implant Enhancements

The application of mussel-inspired polydopamine (PDA) coatings can enhance osteogenic differentiation and osseointegration of various implants. These PDA coatings, when applied to implant surfaces, have demonstrated significant improvements in the adhesion, proliferation, and differentiation of bone marrow stem cells (BMSCs). Additionally, they facilitate the better integration of the implant into bone tissue in vivo. One of the notable outcomes of these coatings is their ability to up-regulate genes associated with osteogenesis, which in turn improves bone regeneration outcomes [17].

Further innovations in implant coatings have seen the incorporation of polydopamine with tannic acid on titanium implants, forming a bioinspired coating. This specific combination (Ti-pTAN) has been found particularly effective in reducing osteoclast activity, a critical factor in minimizing bone resorption. Notably, tannic acid outperforms polydopamine alone in suppressing osteoclast activity. When compared to titanium coated solely with polydopamine (Ti-pDOP), the Ti-pTAN coatings show superior efficacy in reducing osteoclast activity, highlighting their potential for enhanced application at bone interfaces [16].

Progressing beyond single-component systems, the layer-by-layer immobilization of polydopamine-assisted epsilon-polylysine and gum Arabic on titanium has resulted in the creation of composite films. These films not only enhance the antibacterial properties of titanium implants but also improve their osteogenic capabilities. Such dual enhancements make these implants more favorable for clinical outcomes, optimizing both the antibacterial and osteogenic properties crucial for medical titanium implants [21].

In addition, polydopamine-induced nanocomposite coatings consisting of silver/calcium phosphate (Ag/CaP) on titania nanotubes have also been developed. These coatings enhance antibacterial effects and osteointegration, demonstrating improved cellular compatibility and increased alkaline phosphatase (ALP) activity in MG63 cells. The heightened ALP activity observed in these coated implants suggests better osteointegration capabilities compared to uncoated titanium, featuring their potential for enhanced long-term success in implant applications [20].

The integration of PDA-mediated covalent functionalization of collagen on titanium alloys, specifically Ti-6Al-4V, has been shown to be highly promising in improving compatibility with soft tissues. This method involves chemically bonding collagen to the implants via PDA, which enhances cellular adhesion significantly both in vitro and in vivo. This approach not only provides a more stable interface compared to physical adsorption methods but also offers enhanced performance and reliability for soft tissue integration, suggesting its substantial utility in improving implant biocompatibility [6].

Summing up, the application of polydopamine (PDA) coatings has been consistently shown to enhance osteogenic differentiation and osseointegration of implants. This is evidenced by improved adhesion, proliferation, and differentiation of bone marrow stem cells (BMSCs) and the up-regulation of osteogenesis-associated genes [17]. Notably, PDA combined with tannic acid on titanium implants (Ti-pTANs) reduces osteoclast activity more effectively than PDA alone, crucial for minimizing bone resorption and maintaining implant stability [16]. Additionally, composite films created through the layer-by-layer immobilization of PDA-assisted epsilon-polylysine and gum Arabic enhance both antibacterial properties and osteogenic capabilities of titanium implants [21]. PDA-induced nanocomposite coatings of silver/calcium phosphate (Ag/CaP) on titania nanotubes also improve antibacterial effects and demonstrate superior osteointegration, evidenced by increased alkaline phosphatase (ALP) activity in MG63 cells [20]. Furthermore, the PDA-mediated covalent functionalization of collagen on titanium alloys (Ti-6Al-4V) significantly enhances cellular adhesion and integration with soft tissues, offering a more stable interface than physical adsorption methods [6]. Despite these promising results, there remains a need for longitudinal studies to fully understand the long-term in vivo performance and clinical scalability of these innovative coatings. Comparative studies are also necessary to determine the optimal combinations and concentrations of these materials to maximize their efficacy in clinical applications.

Table 3 outlines concerns and limitations associated with the modification of biomaterials using polydopamine, including uncertainties regarding the long-term effects of additives such as tannic acid, the durability of polydopamine coatings across different environments, balancing antibacterial properties with tissue compatibility, evaluating potential cytotoxicity and stability of nanoparticles, and understanding the long-term effects of collagen integration on tissue response.

## 5. Cancer Treatment and Imaging

Polydopamine-based drug delivery systems have demonstrated significant promise in targeting cancer cells more effectively compared to traditional chemotherapy methods. One innovative approach involves using polydopamine-modified mesoporous silica nanoparticles (MSNs) coated with poly(ethylene glycol)-folic acid (PEG-FA). This nanocarrier system, referred to as MSNs@PDA-PEG-FA, is loaded with the chemotherapeutic agent doxorubicin (DOX). The system exploits the pH-sensitive properties of polydopamine to enable controlled and sustained drug release in the acidic microenvironment of tumors, thereby enhancing therapeutic efficacy while minimizing damage to healthy cells [82]. Studies have shown that these nanoparticles achieve a higher targeting efficiency and antitumor efficacy compared to free DOX and non-targeted DOX-loaded nanoparticles, highlighting their potential as a superior drug delivery vehicle for cancer therapy [82].

Furthermore, the integration of polydopamine with photothermal therapy (PTT) capabilities offers additional benefits. Polydopamine-coated nanoparticles grafted with folic acid and poly(ethylene glycol) have been shown to deliver DOX effectively while providing excellent photothermal conversion under near-infrared light. This dual-function system not only targets cancer cells more precisely but also induces tumor cell apoptosis more efficiently than traditional chemotherapy. The combination of chemotherapy and PTT in these nanocarriers results in reduced drug-related cardiotoxicity and improved antitumor efficacy in vivo [83].

The development of multifunctional platforms that combine photothermal therapy, chemotherapy, and immunotherapy further exemplifies the effectiveness of polydopamine-based systems. For instance, nanoparticles co-loaded with DOX and the immune adjuvant imiquimod (R837) and modified with a folate ligand have demonstrated superior tumor suppression and lower recurrence rates compared to single-mode therapies. This synergy between hyperthermia, chemotherapeutic agents, and immune responses highlights the potential of these systems to achieve comprehensive cancer treatment with minimal side effects [84].

Overall, polydopamine-based drug delivery systems represent a significant advancement in cancer therapy by offering targeted, sustained, and multi-modal treatment options that surpass the limitations of traditional chemotherapy. The unique properties of polydopamine, including its biocompatibility, ease of fabrication, and responsiveness to various stimuli, make it an ideal carrier for developing next-generation anticancer therapies [85].

For cancer treatment, a photoelectrochemical (PEC) biosensing interface using polydopamine nanospheres on TiO_2_ nanotube arrays demonstrated enhanced detection capabilities for the breast cancer cell line MDA-MB-231, with a wide linear range from 20 to 2.0 × 10^6^ cells/mL and a low detection limit of 15 cells/mL (S/N = 3) [86]. Additionally, an electrochemiluminescence (ECL) biosensor integrating Au@Mo-PDA hollow spheres achieved the sensitive detection of prostate-specific antigen (PSA) with a linear range from 10 fg/mL to 5 ng/mL and a detection limit of 4.2 fg/mL [87].

Cabazitaxel-loaded pH-responsive PDA nanoparticles (CBZ NP) showed significant improvements in drug delivery against metastatic prostate cancer cells, enhancing cellular uptake, apoptosis markers, and cell cycle arrest [88]. Furthermore, parthenolide-loaded PLGA nanoparticles modified with PDA (PN-PLGA-PDA) demonstrated efficient drug loading with a 96.9% encapsulation efficiency and sustained release, with less than 40% release over 144 h, enhancing cytotoxicity against cancer cells [89].

In tissue engineering, polydopamine-modified PDLLA membranes with sulfonated chitosan (SCS) or phosphorylated chitosan (PCS) significantly enhanced osteogenic differentiation of MC3T3-E1 cells, with PCS showing a superior up-regulation of osteogenic genes [90]. Additionally, DNase I coatings on titanium implants using alternating current electrophoretic deposition (AC-EPD) and PDA coupling significantly increased enzyme activity, reducing biofilm formation of Staphylococcus epidermidis and Pseudomonas aeruginosa for up to 20 h [91]. For bone defect repair, ADSCs-derived exosomes combined with gelatin sponge and PDA (GS-PDA-Exos) scaffolds significantly promoted bone regeneration, with a micro-CT analysis confirming increased new bone formation in femur defect models [62].

**Figure 4:**
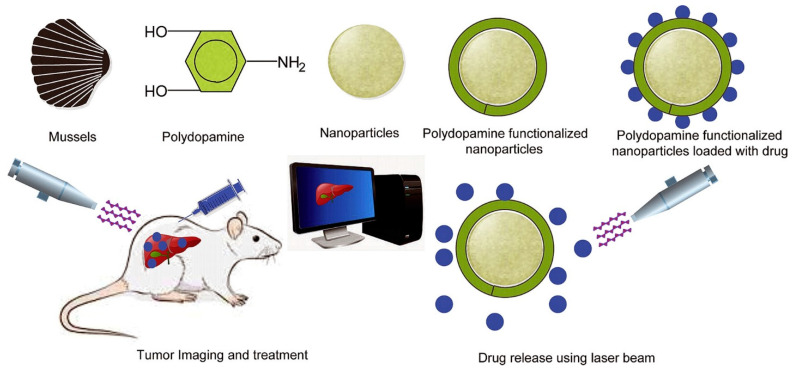
Showing functionalized theranostic nanocarriers with mussel-inspired polydopamine nanoparticles for tumor imaging and chemo-photothermal therapy. Adopted with permission [74].

The field of cancer treatment and imaging is undergoing significant transformation through the integration of bioinspired materials, particularly polydopamine (PDA), which has catalyzed notable advancements in the development of theranostic nanocarriers. A comprehensive review has highlighted the improvements brought by polydopamine-coated nanocarriers in terms of drug loading, release, and therapeutic efficacy, markedly enhancing tumor treatment and imaging capabilities. The dual utility of PDA in both tumor imaging and chemo-photothermal therapy showcases its broad potential in multifaceted cancer management strategies (Figure 4) [74].

Research into the cellular interactions of polydopamine-coated nanoparticles has provided deeper insights into their therapeutic potential. For instance, studies involving polydopamine-coated mesoporous silica nanoparticles within HeLa cells have elucidated the mechanisms of cellular uptake and revealed approaches to improve treatment outcomes. Significant findings from these studies include the targeted drug release to specific cellular organelles and strategies to inhibit exocytosis, which could substantially boost the effectiveness of cancer therapies [92].

Another development is the use of polydopamine-modified 2D iron-immobilized MnPS3 nanosheets. These nanosheets, further enhanced with polyethylene glycol (PEG), demonstrate high photothermal conversion efficiency and potent chemodynamic therapy properties. Their capability for safe excretion post-treatment underscores their viability as a sustainable treatment modality [93].

In addressing triple-negative breast cancer, a particularly aggressive and treatment-resistant form of cancer, precision combination therapies employing biomimetic polydopamine nanostructures have been introduced. These nanostructures facilitate controlled, localized drug release activated by photothermal stimuli. The design allows the nanoparticles to collapse under tumor-localized heat, directly releasing drugs into the tumor site, thus minimizing systemic side effects and maximizing treatment efficacy [94].

**Figure 5:**
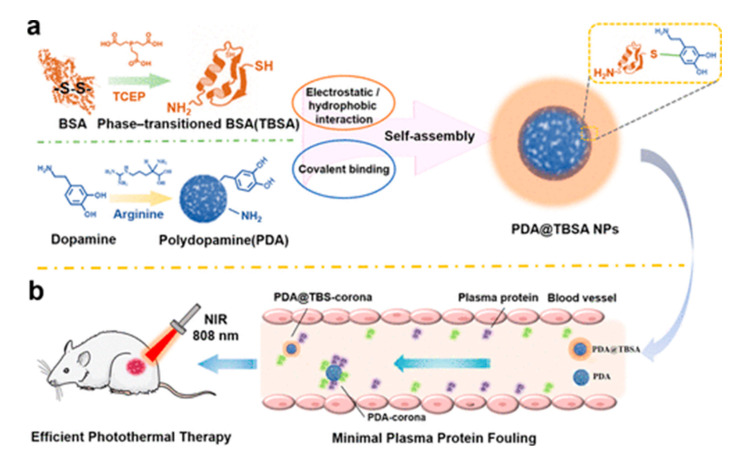
A schematic illustration showing (**a**) phase-transitioned albumin biomimetic nanocorona (TBSA) nanoparticles with PDA formed by the BSA-derived amyloid through the hydrophobic aggregation near the isoelectric point and the rupture of disulfide bonds by tris(2-carboxyethyl) phosphine. (**b**) PDA@TBSA’s ability to pass the blood–brain barrier to produce photothermal antitumor. Reprinted (adapted) with permission from Han, et al. (2022). Copyright 2022 American Chemical Society [95].

Further advancements include the engineering of polydopamine nanoparticles to minimize plasma protein fouling while simultaneously enhancing photothermal therapy. These nanoparticles are modified with amyloid proteins, significantly reducing undesirable interactions with plasma proteins, thereby improving therapeutic outcomes. The resultant PDA@TBSA nanoparticles greatly enhance tumor residence time and enrichment, which are critical in reducing plasma protein adsorption and boosting the effectiveness of photothermal therapy (Figure 5) [95].

Moreover, a biomimetic multifunctional nanoplatform using polydopamine-wrapped gold nanoparticles has been employed for the multilayer imaging of cancer biomarkers. This technology has been instrumental in distinguishing cancer-derived extracellular vesicles, thereby advancing the high-throughput imaging of cancer biomarkers. This approach has shown considerable promise in enhancing the diagnostics of hepatocellular carcinoma, facilitating the detection of cancer biomarkers at the single-vesicle level [96].

PDA-based drug delivery systems have demonstrated superior targeting and therapeutic efficacy compared to traditional chemotherapy, showcasing significant advancements in cancer treatment. For instance, PDA-modified mesoporous silica nanoparticles (MSNs@PDA-PEG-FA) enable controlled drug release in the acidic tumor microenvironment, thereby enhancing the antitumor effects of doxorubicin (DOX) while minimizing harm to healthy cells [82]. The integration of PDA with photothermal therapy (PTT) has further shown promise; PDA-coated nanoparticles combined with folic acid and poly(ethylene glycol) not only effectively deliver DOX but also induce tumor cell apoptosis through photothermal conversion, thus improving overall antitumor efficacy [83]. Multifunctional platforms that integrate chemotherapy, PTT, and immunotherapy, such as nanoparticles co-loaded with DOX and the immune adjuvant imiquimod (R837), demonstrate superior tumor suppression and reduced recurrence rates [84]. Noteworthy is the development of PDA-modified 2D iron-immobilized MnPS3 nanosheets, which provide high photothermal conversion efficiency and potent chemodynamic therapy properties, with safe post-treatment excretion [93]. For triple-negative breast cancer, biomimetic PDA nanostructures offer precision combination therapies with controlled, localized drug release, enhancing treatment efficacy and minimizing systemic side effects [94]. Despite these advances, further comparative studies are necessary to optimize material combinations and concentrations for maximum therapeutic outcomes. Long-term in vivo studies are essential to evaluate the clinical scalability and long-term performance of these innovative PDA-based systems. Moreover, strategies to reduce plasma protein fouling, as demonstrated by PDA@TBSA nanoparticles, are needed to improve tumor residence time and enrichment, thus boosting photothermal therapy effectiveness [95].

Table 4 highlights concerns and limitations associated with the use of polydopamine-modified nanoparticles for cancer therapy, including systemic toxicity, precise targeting, unintended cellular effects, resistance, efficient excretion, safety, potential toxicity, long-term biodegradation, specificity in complex biological samples, accuracy, and long-term safety of amyloid proteins.

## 6. Nanotechnology Applications

In the medical arena, PDA’s properties are particularly noteworthy. Its excellent adhesion, light absorption, and heat conversion capabilities make it an ideal candidate for tumor ablation and other therapeutic strategies. The adaptability of PDA in forming coated surfaces, nanoparticles, and composites extends its utility in medical applications, ranging from photothermal therapy to sophisticated drug delivery systems. This adaptability underlines PDA’s potential to transform medical treatments [7].

Further expanding on the utility of polydopamine, research has focused on the control and fluorescence labeling of melanin-mimetic polydopamine nanoparticles (MMNPs). These nanoparticles are synthesized with specific attributes, such as controlled size and fluorescence, making them suitable for detailed biomedical imaging and drug delivery. The precise control over particle diameter, surface charge, and fluorophore loading enhances their capabilities for detailed intracellular imaging, demonstrating the advanced engineering of nanomaterials for specialized medical applications [75].

The integration of inorganic nanostructures with polydopamine-derived carbon also showcases the adaptability of PDA in various technological applications. PDA serves as a carbon source, integrating with inorganic structures to yield composite nanostructures. These composites are tailored for use in diverse sectors, such as energy and electronics, illustrating the ease with which the structure and morphology of these nanostructures can be manipulated to enhance their functionality [97].

Moreover, the application of mussel-inspired polydopamine for coating nanoparticles reveals its stability and biocompatibility, particularly in vivo. Gold nanoparticles coated with polydopamine demonstrate remarkable stability and low cytotoxicity, enhancing their suitability for intracellular delivery. These PDA-coated nanoparticles have been shown to remain stable in liver and spleen cells for at least six weeks, without causing notable histological toxicity, further emphasizing their potential for safe and effective use in live organisms [51].

In targeted cancer therapy, the polydopamine-enabled surface functionalization of gold nanorods has shown promising results. These nanorods, coated with polydopamine and functionalized with antibodies, are specifically designed for targeted photothermal cancer therapy. The functionalization allows the nanorods to bind to specific cancer cells, effectively targeting and destroying them under light activation. This method has been proven to significantly enhance the precision and efficacy of photothermal therapy, providing a focused approach to cancer treatment [57].

Polydopamine’s versatility extends to the surface modification of nanodiamonds, where it has been used to reduce silver ions to nanoparticles. This bioinspired modification not only enhances the functionality of the nanodiamonds by allowing them to anchor various chemical species but also increases the size and number of silver nanoparticles as the concentration of silver ions increases. Such modifications contribute to the multifunctional capabilities of nanodiamonds, expanding their application in various nanotechnology sectors [98].

In another innovative application, polydopamine has been employed for the surface engineering of magnetic nanoparticles via a metal-free photoinduced electron transfer-atom transfer radical polymerization process. This integration of polydopamine chemistry allows for the creation of functional polymer brushes on the nanoparticles, which have shown improved hydrophilicity, dispersibility, and enhanced binding to uranyl ions. These functionalities make the nanoparticles particularly useful in biomedicine and environmental engineering, demonstrating the broad applicability of polydopamine-modified materials [45].

The mussel-inspired functionalization of graphene using polydopamine illustrates another avenue for creating antibacterial materials. By incorporating silver nanoparticles into polydopamine–graphene nanosheets, a mild and environmentally friendly method was developed that ensures uniform dispersion of the nanoparticles. These functionalized nanosheets exhibit strong antibacterial properties, inspired by the natural adhesive proteins of mussels. This approach not only provides an effective means of combating bacterial growth but also underscores the potential for developing new antibacterial materials [23].

**Figure 6:**
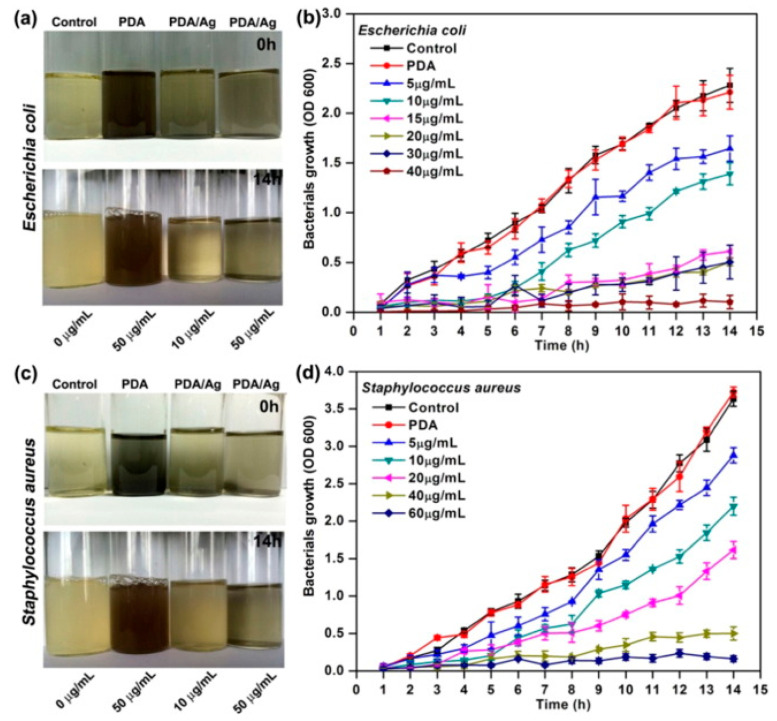
Luria–Bertani (LB) liquid medium used in controlled experiments to measure the growth of bacterial using optical density (O.D.) at a wavelength of 600 nm. The starting point was noted when the PDA/Ag nanocomposite particles were added to the LB bacterium suspension. (**a**) Photograph taken with samples incubated for 0 h and 14 h with LB liquid medium containing 10 and 50 μg/mL: control, PDA, and PDA/Ag nanocomposite particles, with *Escherichia coli* (*E. coli*) to evaluate the antibacterial activities. (**b**) Photograph taken with samples incubated for 0 h and 14 h with LB liquid medium containing 10 and 50 μg/mL: control, PDA, and PDA/Ag nanocomposite particles, with *Staphylococcus aureus* (*S. aureus*) to evaluate the antibacterial activities. (**c**) Measurement of OD with different concentrations of the PDA/Ag nanocomposite particles (5–40 μg/mL) and pure PDA particles (40 μg/mL) with *E. coli* at 0 h to 14 h. (**d**) Measurement of OD with different concentrations of the PDA/Ag nanocomposite particles (60 μg/mL) and pure PDA particles (60 μg/mL) for *S. aureus* at 0 h to 14 h. Adopted with permission [5].

The bioinspired creation of polydopamine/silver (PDA/Ag) nanocomposite particles represents a notable breakthrough in antibacterial materials. By leveraging the natural catechol and amine groups of polydopamine to reduce silver, a green synthesis method was established. These nanoparticles demonstrate potent antibacterial properties against common pathogens, such as Escherichia coli and Staphylococcus aureus, while not significantly harming human cells. This combination of antibacterial effectiveness and biocompatibility highlights the potential of PDA/Ag nanoparticles for medical applications where preventing infections is essential (Figure 6) [5].

In another study, the electropolymerization of polydopamine on mesoporous silica films was explored with an emphasis on regulating ion transport in applications such as water management and catalysis. This research revealed that the number of electropolymerization cycles and the properties of the films significantly influence the distribution of PDA within the mesoporous structure, affecting the control of ionic transport. This process highlights the potential of polydopamine coatings to enhance the functionality of mesoporous materials in various industrial and environmental applications [99].

The versatility of polydopamine-like coatings has been extended further by developing a method to copolymerize catechol and diamine. This approach led to the creation of a functional primer coating applicable to various substrates to confer hydrophobic properties. The successful application of this coating demonstrates its tunable reactivity and enhanced hydrophobicity, making it a valuable tool for surface functionalization across a broad range of materials and uses [100].

The biomimetic oligopeptide modification of polydopamine surfaces has been studied for its potential in tissue engineering, particularly for vascular prostheses. By functionalizing polydopamine surfaces with peptides that control cell adhesion and growth, researchers have identified combinations such as RGD and collagen (Col) that are particularly effective in enhancing cell adhesion stability under dynamic loads. This approach shows promise for the development of vascular grafts that require durable, bioactive surfaces to support cell integration and function [101].

The development of antifouling, high-flux nanofiltration membranes using polydopamine represents a notable advancement in water treatment technology. These polydopamine-coated ultrafiltration membranes have been specifically modified to enhance separation performance while incorporating antifouling properties. In testing, these membranes have demonstrated excellent antifouling capabilities, characterized by a high flux recovery ratio and minimal flux decline, making them highly effective for maintaining performance over prolonged periods in water purification processes [102].

In the field of isotope separation, well-defined functional mesoporous silica/polymer hybrids have been created using bioinspired polydopamine chemistry integrated with atom transfer radical polymerization (ATRP). These hybrids have been specifically used for the selective separation of lithium isotopes. A detailed study was conducted to evaluate the adsorption behavior and the effects of environmental factors on the efficiency of lithium separation. This research underscored the potential of these hybrids to contribute significantly to the field of material science, particularly in applications requiring precise molecular separations under varying environmental conditions [49].

The synthesis of magnetically separable and recyclable hybrid microspheres for catalytic applications further illustrates the utility of polydopamine. These Fe_3_O_4_-polydopamine hybrid hollow microspheres exhibit peroxidase-like activity and improved stability, making them useful in biocatalysis and environmental monitoring. Their ability to enhance stability and catalytic efficiency in oxidation reactions offers valuable insights into the design of new catalysts that can be easily separated and reused, reducing both cost and environmental impact [48].

In the context of energy conversion, polydopamine plays a crucial role in the field of artificial photosynthesis as an electron gate that significantly enhances the efficiency of photoinduced electron transfer and photochemical water oxidation. This functionality highlights the potential of polydopamine to increase the efficiency of energy conversion processes in artificial photosynthesis systems, making it a valuable component for sustainable energy technologies [103].

Additionally, the development of copper-coated liquid-crystalline elastomers via polydopamine adhesion has shown potential for creating conductive properties useful in applications such as thermoreversible actuators. Despite some loss of conductivity over thermal cycling, these materials have maintained structural integrity without delamination after 25 thermal cycles. This durability suggests the potential for these elastomers to be used in applications where mechanical flexibility and electrical conductivity are required, albeit with considerations for managing conductivity changes with thermal cycling [104].

In summary, polydopamine (PDA) is noted for its exceptional adhesion, light absorption, and heat conversion properties, making it highly suitable for medical applications, like tumor ablation and drug delivery systems [7]. For instance, PDA-modified mesoporous silica nanoparticles demonstrate controlled size and fluorescence, which are crucial for advanced biomedical imaging and drug delivery [75]. Beyond medical uses, PDA’s role as a carbon source in composite nanostructures showcases its adaptability in energy and electronics [97]. In vivo studies on PDA-coated gold nanoparticles reveal significant stability and low cytotoxicity, essential for effective intracellular delivery [51]. Additionally, PDA-functionalized gold nanorods enhance the precision and efficacy of photothermal therapy in targeted cancer treatments [57]. PDA’s versatility extends to the surface modification of nanodiamonds, improving their functionality by anchoring various chemical species [98]. Innovations like the metal-free photoinduced electron transfer-atom transfer radical polymerization process enhance the hydrophilicity and binding capabilities of magnetic nanoparticles, demonstrating PDA’s broad applicability [45]. The antibacterial properties of PDA/Ag nanocomposite particles offer effective an antibacterial action without significant harm to human cells [5]. However, there are gaps in optimizing these applications for clinical and industrial scalability. Comparative studies to standardize PDA concentrations and combinations, as well as long-term in vivo studies, are necessary to fully understand their practical implications and performance.

Table 5 outlines concerns and limitations across various applications of polydopamine modification, encompassing issues such as reproducibility, immune reactions, toxicity, stability, off-target effects, resistance, environmental impact, scalability, and long-term performance.

## 7. Tissue Engineering and Cell Culture Technologies

In tissue engineering and cell culture technologies, the use of polydopamine (PDA) has significantly enhanced the functionality and applicability of various materials, demonstrating its transformative impact across diverse biomedical applications.

**Figure 7:**
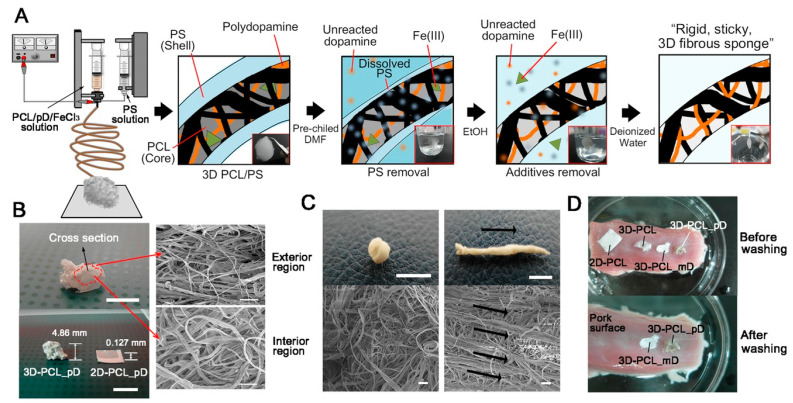
(**A**) Schematic illustration of the methodology to fabricate rigid, sticky, 3D fluffy PCL fibrous sponges containing pDA networks (3D-PCL_pD). (**B**) Digital images showing the physical morphology of 3D-PCL_pD sponges (scale bar: 5 mm) and SEM images of the 3D-PCL_pD exterior and interior region (scale bar: 10 μm). (**C**) Digital images showing the moldable properties of the resulting pDA-containing PCL fibers (scale bar: 10 mm) and the exterior surfaces (SEM images) after manual deformation (scale bar: 10 μm). The arrows indicate the direction of deformation of the pDA-containing PCL fibers. (**D**) Digital images of the sticky characteristics of the resulting 3D-PCL sponges fabricated during the same processing time containing dopamine or pDA components onto a wet porcine surface after. Adopted with permission [39].

One key application involves the structural enhancement of 3D electrospun polycaprolactone sponges through polydopamine integration. This modification not only increases the rigidity and stickiness of these sponges but also promotes robust biomolecule immobilization, crucial for applications in both tissue engineering and targeted drug delivery systems. The iron ion-induced oxidation process involved in this enhancement also aids in the stable attachment of biomolecules on wet organ surfaces, broadening the medical applicability of these sponges (Figure 7) [39].

Further advancements in polydopamine application include its role in improving the adhesion of preosteoblasts on 3D polycaprolactone (PCL) scaffolds. By applying a polydopamine coating followed by a mineralization process, the originally hydrophobic surfaces of these scaffolds are rendered hydrophilic, significantly enhancing preosteoblast infiltration and adhesion. This modification not only improves cell compatibility within the scaffolds but also promotes better outcomes in bone regeneration projects, making it a widely applicable technique in bone tissue engineering [107].

In periodontal tissue engineering, polydopamine-containing, hierarchically patterned membranes have been developed to optimize the adhesion of therapeutic proteins and cells. These nanofibrous membranes accelerate osteogenic differentiation and enhance hydroxyapatite mineralization, contributing to successful tissue and bone repair in rat models. The multifunctional platform provided by polydopamine highlights its significant value in improving bioactivity and structural support in tissue engineering applications [73].

The development of polydopamine-induced hydroxyapatite coatings on polyamide implants marks an advancement in enhancing osteogenic induction. This approach not only improves bone integration both in vitro and in vivo but also increases the bioactivity of the scaffolds, potentially accelerating the osseointegration process. Key enhancements such as improved hydrophilicity and surface roughness contribute to increased bone formation, underlining the importance of polydopamine in the development of effective bone implants [68].

Polydopamine’s application extends to enhancing the functionality of biomaterials used in stem cell technology. For instance, polydopamine-coated polycaprolactone/gelatin (PCL/gelatin) nanofibrous membranes significantly enhance the adhesion and osteogenic differentiation of adipose stem cells. This improvement facilitates the construction of 3D complex tissue structures, making these membranes highly suitable for tissue engineering applications [108].

In reconstructive surgery, specifically for pelvic organ prolapse repair, a polypropylene mesh modified with small intestinal submucosa via a polydopamine coating has shown enhanced biocompatibility. In vivo tests demonstrate reduced fibroplasia and the decreased expression of pro-inflammatory macrophages, along with significantly lowered levels of the inflammatory cytokines IL-1beta and IL-6, suggesting improved clinical outcomes in pelvic reconstruction by mitigating inflammation and enhancing tissue integration [36].

The stabilization of mesenchymal stem cell adhesion and multipotency on polydimethylsiloxane (PDMS) surfaces has been achieved through polydopamine coating. This modification enhances the compatibility of PDMS for bone marrow stromal cell culture, facilitating prolonged cell adhesion and preserving cell multipotency, crucial for mechanobiological studies. The bio-modified PDMS surfaces provide a stable and conducive environment for the growth and differentiation of mesenchymal stem cells, broadening the scope of their applications in biomedical research [109].

Additionally, polydopamine-mediated surface modification has been effectively utilized for engineering scaffolds that support the proliferation and differentiation of human neural stem cells. This technique offers a simple and efficient method for enhancing scaffold performance, providing a safer alternative to animal-derived materials for stem cell-based therapies. The modified scaffolds facilitate enhanced differentiation and proliferation of human neural stem cells, representing a significant step forward in developing ethical and effective materials for neural stem cell engineering [110].

Polydopamine coatings also play a crucial role in enhancing the performance of biomedical devices, particularly in vascular health applications. For instance, the application of polydopamine coatings to stainless steel stents significantly improves their hemocompatibility, enhancing endothelial cell function while simultaneously reducing the proliferation of smooth muscle cells, crucial for preventing restenosis. This modification leads to improved adhesion, proliferation, migration, and bioactivity of human umbilical vein endothelial cells (HUVECs), while reducing proliferation in human umbilical artery smooth muscle cells (HUASMCs) on stainless steel surfaces. These improvements suggest that polydopamine-coated stents could offer more effective and safer options for re-endothelialization in vascular devices [111].

A polydopamine-decorated polycaprolactone-co-lactide (PLCL) conduit has been designed for nerve repair to induce synergistic effects of electrical stimulation and topological morphology for peripheral nerve regeneration. This innovative conduit, featuring a conductive, biodegradable structure with micro-patterned surfaces, has shown promising results in facilitating peripheral nerve regeneration. In a rat sciatic nerve crush model, the conduit promoted significant improvements in neuronal expression and myelin sheath growth, leading to enhanced functional recovery. This approach presents an innovative and effective strategy for treating peripheral nerve injuries (PNIs) by harnessing the combined effects of electrical stimulation and physical cues to support nerve healing [35].

Further advancements in neural technology involve the development of mechanically stable platinum black microelectrodes using a mussel-inspired polydopamine adhesive. These microelectrodes are designed for neural signal recording and have demonstrated low impedance and high mechanical stability under conditions such as ultrasonication. The low impedance and high charge injection limits of these electrodes enhance their performance in neural recording applications, offering improved durability and reliability in capturing neural signals [34].

The synthesis of polydopamine nanospheres has introduced a novel method for biomimetic hydroxyapatite formation. These nanospheres are created through spontaneous oxidative polymerization and serve as templates for the mineralization of hydroxyapatite, aiming to enhance biocompatibility and bioactivity for applications in tissue engineering. The resulting hydroxyapatite has demonstrated successful compatibility with fibroblasts and has supported cell spreading and viability above 90%. This indicates a strong potential for these polydopamine nanospheres to be used in biomedical applications where enhanced biomimetic mineralization is desired, such as in bone regeneration and repair [47].

In summary, the integration of polydopamine (PDA) applications in tissue engineering and cell culture technologies reveal significant advancements and pinpoint crucial gaps in the literature. The use of PDA in 3D electrospun polycaprolactone (PCL) sponges enhances rigidity, biomolecule immobilization, and tissue engineering applications [39]. PDA coatings on PCL scaffolds improve hydrophilicity, preosteoblast adhesion, and bone regeneration [107]. In periodontal tissue engineering, PDA-enhanced membranes boost protein and cell adhesion, accelerating osteogenic differentiation and mineralization [73]. PDA-induced hydroxyapatite coatings on polyamide implants enhance osteogenic induction and bone integration in vitro and in vivo [68]. Additionally, PDA-coated PCL/gelatin nanofibrous membranes significantly improve adipose stem cell adhesion and differentiation, aiding complex 3D tissue structure construction [108]. In reconstructive surgery, PDA-modified polypropylene meshes enhance biocompatibility and tissue integration while reducing inflammation [36]. PDA coatings on polydimethylsiloxane (PDMS) surfaces stabilize mesenchymal stem cell adhesion and multipotency, benefiting mechanobiological studies [109]. Furthermore, PDA-modified scaffolds support human neural stem cell proliferation and differentiation, offering an ethical alternative to animal-derived materials [110]. In vascular health, PDA-coated stainless-steel stents improve hemocompatibility and endothelial cell function, reducing restenosis risk [111]. PDA-decorated polycaprolactone-co-lactide (PLCL) conduits show promising peripheral nerve regeneration results [35]. Despite these advancements, further research is needed to standardize PDA application protocols, optimize material combinations, and conduct long-term in vivo studies to fully achieve PDA’s clinical potential.

Table 6 summarizes concerns and limitations associated with polydopamine modification across various tissue engineering and medical device applications. It highlights potential challenges. such as scale-up production, long-term stability, viability and functionality of cells in 3D scaffolds, mechanical integrity of hybrid meshes, consistency in properties, variability in coating effectiveness, durability of microelectrodes, stability and performance of biomimetic mineralization, and risks associated with degradation products.

## 8. Controlled Delivery Systems

The field of innovative drug delivery systems is experiencing a shift with the integration of polydopamine (PDA)-based nanotechnology, enhancing the efficacy and targeting of therapeutic agents across various medical disciplines. Polydopamine (PDA) has shown significant promise in enhancing drug release kinetics and patient compliance compared to traditional delivery systems.

The unique properties of PDA, including its pH sensitivity and biocompatibility, enable it to serve as an effective drug carrier, providing controlled and sustained drug release. For example, a study involving magnetic molecularly imprinted polydopamine (DOX-IP) demonstrated that the application of an external magnetic field significantly improved the concentration of doxorubicin in tumor tissues, leading to enhanced tumor growth suppression in a breast adenocarcinoma mouse model. This targeted delivery system minimizes unwanted side effects and increases drug efficacy [112]. Similarly, PDA-coated mesoporous silica nanoparticles (MSNs) have been used to achieve controlled release of cisplatin. The PDA layer, combined with graphene oxide (GO) wrapping, facilitated a sustained release of the drug, improved dispersibility, and enhanced cellular uptake. This multifunctional system demonstrated high cytotoxicity against cancer cells while maintaining low toxicity in its unloaded form, thereby showing potential for improved patient compliance and therapeutic outcomes [113]. Another approach involves the use of PDA capsules conjugated with folic acid, which target folate-receptor-overexpressing cancer cells. These polycapsules not only enhance cell uptake through receptor mediation but also provide pH-responsive drug release, increasing the reactive oxygen species (ROS) levels in targeted cells. Such precise targeting and controlled release mechanisms promise significant improvements in patient compliance by reducing off-target effects and side effects commonly associated with conventional chemotherapy [114]. Moreover, PDA’s capacity to form stable conjugates with drugs such as bortezomib (BTZ) further exemplifies its utility in drug delivery. In an alginate-conjugated polydopamine system, BTZ is released in a pH-dependent manner, tailored to the acidic environment of cancer tissues. This selective release mechanism follows a non-Fickian diffusion model, highlighting PDA’s potential to provide consistent therapeutic levels of drugs while minimizing dosing frequency [115]. Additionally, PDA nanoparticles have been explored for their ability to control the release of gentamicin. In situ polymerization techniques allowed for the loading of gentamicin into PDA nanoparticles, with release kinetics showing a significant release at lower pH levels due to the protonation of the amine groups in PDA. This pH-responsive release mechanism supports the sustained release of the antibiotic over several days, enhancing its therapeutic efficacy and patient adherence [116]. Overall, PDA-based drug delivery systems offer substantial improvements over existing methods by providing controlled, sustained, and targeted drug release, thereby enhancing therapeutic efficacy and patient compliance.

### Enhancements in Drug Release Kinetics and Patient Compliance Using Polydopamine-Based Delivery Systems

Polydopamine (PDA) has shown substantial potential in enhancing drug release kinetics through various strategies. For instance, dopamine-modified titanium nanotube arrays (TNTs) significantly improved ibuprofen (IBU) loading and prolonged drug release. The PDA modification enhanced biomineralization, which is critical for bone implant therapies [117]. Additionally, innovative hybrid inorganic–organic drug multilayer coating processes have been developed to improve drug release time. A study on PDA-coated nanostructured titanium hydroxide (TiOH) surfaces demonstrated prolonged drug release and enhanced antibacterial performance, showing promise for improving the longevity and bioactivity of Ti-based biomaterials [118].

PDA can induce drug release through stimuli such as near-infrared (NIR) light irradiation and pH changes. For example, a laser-responsive antimicrobial nanocomposite hydrogel combining PDA nanoparticles with a peptide amphiphile enabled laser-induced drug release, preventing bacterial growth and providing a significant advance in smart materials for controlled drug delivery applications [119]. Another study developed a pH-responsive hydrogel system (PHG-PDA) incorporating PDA fibers into pullulan hydrogel. This system achieved higher release rates at acidic pH, beneficial for targeting tumor environments [120].

PDA-based drug delivery systems have been designed to improve patient compliance by enabling targeted, controlled, and sustained drug release, reducing dosing frequency, and minimizing side effects. For instance, an active targeting multi-responsive drug delivery platform was developed where doxorubicin hydrochloride (DOX) was loaded onto PDA and coated with cystamine-modified hyaluronic acid (HA-Cys). This nanoplatform exhibited photothermal conversion, tumor-targeting, and pH/redox/NIR-sensitive drug release capabilities, enhancing chemotherapy efficacy and reducing systemic toxicity [121].

PDA’s versatility and biocompatibility make it an excellent material for drug delivery applications. PDA can be easily functionalized to improve cellular uptake, blood circulation, and drug loading. Understanding the connections between PDA nanosystems’ properties and drug delivery mechanisms is pivotal for designing new nanocarriers tailored to specific applications. This comprehensive understanding enables the creation of drug delivery systems that offer better control over drug release and improved patient outcomes [122].

The advancements in PDA-based drug delivery systems, including controlled and stimuli-responsive drug release, improved drug loading, and enhanced biocompatibility, contribute to better drug release kinetics and patient compliance compared to the existing delivery systems. These systems offer targeted, sustained, and efficient drug delivery, minimizing side effects and reducing dosing frequency, thereby improving patient adherence to treatment protocols.

A new advancement is the development of glucose–polydopamine-coated nanoparticles designed to facilitate the efficient crossing of the blood–brain barrier (BBB). This specialized coating significantly boosts the targeting and transcytosis capabilities of nanoparticles in brain capillary endothelial cells, thereby improving the delivery of therapeutic agents directly to brain tissue. Demonstrated in an orthotopic glioblastoma model, these nanoparticles showed high brain tissue uptake, marking a major step forward in potentially transforming treatments for neurological conditions and brain tumors [123].

In dermatology, the application of polydopamine coatings on nonporous and mesoporous nanoparticles has been extensively studied for skin drug delivery. Research comparing these two nanoparticle types revealed that mesoporous nanoparticles, with their structured void spaces, exhibit superior drug loading capacity, effective radical scavenging abilities, and enhanced penetration into deeper skin layers. This enhanced performance is crucial for delivering therapeutic agents more effectively within the skin, promising advancements in treatments for various skin conditions and enhancing cosmetic formulations [124].

The versatility of polydopamine coatings extends to vascular health, where they have been used to immobilize gene complex nanoparticles on substrates to promote endothelial cell proliferation over smooth muscle cells. This application is particularly significant in vascular implants, where localized delivery of growth factor-encoding genes can improve implant functionality and integration. The successful immobilization of these gene complexes has demonstrated potential in promoting specific cellular activities essential for the successful operation of vascular grafts and stents [93].

Further investigations into the synthesis and mechanical properties of polydopamine nanoparticles have led to a PEGylation strategy that enhances their stability and functionality. This research has provided critical insights into optimizing nanoparticle synthesis for drug delivery applications, particularly emphasizing the relationship between nanoparticle size and their mechanical properties. The findings highlight that smaller nanoparticles exhibit greater elasticity, with PEGylation significantly improving their stability under physiological conditions, thus enhancing their utility as drug carriers [46].

The use of polydopamine-coated silicon nanowires for siRNA delivery has shown promising results in enhancing cellular uptake and gene silencing efficacy. The polydopamine coating improves siRNA attachment, facilitating effective gene knockdown and aiding in membrane perturbation necessary for successful transmembrane delivery. This application has demonstrated enhanced cell adhesion and siRNA anchoring, leading to efficient gene silencing and indicating significant potential in gene therapy and targeted treatments [125].

The unique properties of polydopamine (PDA), including its pH sensitivity and biocompatibility, make it an exceptional carrier for controlled and sustained drug release, significantly enhancing therapeutic efficacy and patient compliance across various medical fields. Research has demonstrated the diverse applications of PDA-based systems in targeted cancer therapy, dermatology, vascular health, and antibiotic delivery. For instance, magnetic molecularly imprinted PDA (DOX-IP) systems improve doxorubicin concentration in tumors under an external magnetic field, enhancing tumor suppression with minimal side effects [112]. Similarly, PDA-coated mesoporous silica nanoparticles (MSNs) with graphene oxide wrapping facilitate sustained cisplatin release and improved cellular uptake, showing high cytotoxicity against cancer cells [113]. Additionally, PDA capsules conjugated with folic acid provide targeted, pH-responsive drug release, increasing reactive oxygen species (ROS) levels in targeted cells and reducing off-target effects [114]. The pH-responsive release of gentamicin from PDA nanoparticles supports sustained antibiotic release, enhancing therapeutic efficacy [116]. Furthermore, glucose-PDA-coated nanoparticles significantly improve therapeutic agent delivery to brain tissue, crossing the blood–brain barrier (BBB) [123], while in dermatology, PDA coatings on mesoporous nanoparticles enhance drug loading capacity and skin penetration [124]. Despite these advancements, optimizing PDA application protocols, understanding long-term in vivo performance, and standardizing material combinations remain challenges.

Table 7 highlights concerns and limitations related to the use of polydopamine-based drug delivery systems, including the potential toxicity of nanoparticles, long-term effects of chronic exposure, risks associated with gene therapy such as off-target effects and immune responses, challenges in achieving consistent nanoparticle properties, and potential cytotoxicity from high aspect ratio nanomaterials.

## 9. Sensing, Diagnostics, and Analytical Techniques

Polydopamine (PDA) in the field of sensing, diagnostics, and analytical techniques, offers substantial enhancements to biosensing capabilities and surface modification technologies. Its adaptability and functional properties allow for meaningful improvements across various diagnostic platforms.

One pivotal area where PDA has demonstrated its utility is in the enhancement in biosensing surfaces, particularly for applications involving Raman scattering. By confining nanoparticles within a PDA matrix on biosensing surfaces, researchers have been able to create highly active surfaces for enhanced Raman scattering. This application not only showcases the adaptability of PDA in biosensing but also its effectiveness in boosting the sensitivity and functionality of biosensors [29].

In another intervention, PDA-coated membranes have been employed to develop a colorimetric sensor for detecting hemoglobin A1c (HbA1c) levels in the blood, utilizing immobilized recombinant fructosyl peptide oxidase. This sensor maintains 85% of its activity even after repeated use, demonstrating enhanced enzyme immobilization and stability. This durability is crucial for providing a reliable and repeatable detection of HbA1c levels, offering a valuable tool in the management of diabetes [34].

Expanding its applications in medical diagnostics, PDA has been used to improve antibody immobilization on surfaces. By electrochemically enhancing a PDA thin film, antibody loading was increased by 27%, significantly boosting the sensitivity of immunoassays for detecting prostate-specific antigen. This advancement enhances diagnostic capabilities and highlights the potential of PDA to improve the performance of various immunoassay platforms [28].

PDA-coated nanospheres decorated with silver have also been utilized in the development of a thrombin assay. This setup enhances electrochemiluminescent signals, providing high sensitivity and specificity for thrombin detection. With a detection limit of 0.35 fmol/L and a linear response range from 1.0 fmol/L to 5.0 nmol/L, the assay demonstrates clinical applicability and potential for broader use in medical diagnostics [35].

Moreover, the exploration of PDA-coated zinc oxide nanorods in photoelectrochemical immunoassays has revealed the dual functionality of PDA. Not only does it enhance charge separation, but it also facilitates antibody attachment, creating a reliable label-free detection platform. This application successfully detected an antibody–antigen model with a detection limit of 10 pg/mL, illustrating the broad potential of PDA-coated materials in enhancing immunoassay techniques and other diagnostic applications [32].

In environmental sensing, a sensor developed using zinc oxide nanorods coated with PDA and gold nanoparticles has significantly enhanced the photocurrent response for detecting amyloid-beta, crucial for early Alzheimer’s disease diagnosis. The sensor demonstrated excellent selectivity, stability, and reproducibility, with a detection limit as low as 0.26 pg/mL and a wide linear range from 1 pg/mL to 100 ng/mL, showcasing its high sensitivity and potential for clinical applications [30].

In electrochromatography, a novel capillary column coated with PDA and subsequently attached to zeolitic imidazolate framework-8 has improved separation efficiency and interaction with analytes, thereby enhancing chromatographic performance. The column achieved a high separation efficiency with theoretical plate numbers up to 1.9 × 10^5^ N for catechol, demonstrating its effectiveness in chromatographic applications [26].

Additionally, in capillary electrophoresis, a review of PDA surface modifications highlighted the challenges and strategies for achieving better reproducibility and operability in polydopamine-coated capillaries. This discussion underscores the significant advances in the application of PDA in capillary electrochromatography, emphasizing its role in modifying capillary surfaces to introduce various chromatographic mechanisms [2].

In food safety testing, a surface-enhanced Raman scattering (SERS) sensor developed using titanium dioxide (TiO_2_) nanorods coated with PDA and silver enhanced electron transfer and enriched target dyes, enabling the effective detection of illegal food dyes, like rhodamine B and crystal violet. The sensor demonstrated individual and duplex detection capabilities with low detection limits, highlighting its potential for ensuring food safety [127].

Furthermore, a highly selective fluorescence sensor for metronidazole was created using PDA molecularly imprinted polymers on an iron-based metal–organic framework. This sensor achieved a low detection limit and wide linear range with minimal interference from other substances, proving effective even in complex samples like milk and serum. The sensor exhibited a detection limit of 53.4 nM and a linear range from 1 to 200 µM, demonstrating negligible interference from common substances [33].

In addition, PDA-modified reduced graphene oxides (PDRGOs) have been utilized for enhanced biosensing applications. The PDA-coated graphene oxide served as a scaffold to anchor gold nanoparticles, enhancing the electrochemical properties and catalytic activity towards the oxidation of NADH. This setup was further developed into a sensitive alcohol biosensor, showcasing enhanced electrocatalytic activity and low potential operation suitable for sensitive alcohol detection [31].

Various studies have demonstrated PDA’s ability to improve sensor sensitivity and reliability. For instance, the PDA matrix confinement of nanoparticles enhances surfaces for Raman scattering, boosting biosensor sensitivity [29]. PDA-coated membranes used in colorimetric sensors for HbA1c detection retain a high enzyme activity, critical for diabetes management [34]. Electrochemically enhanced PDA films improve antibody immobilization, increasing immunoassay sensitivity for prostate-specific antigen detection [28]. PDA-coated nanospheres with silver show a high clinical applicability in thrombin assays, with a detection limit of 0.35 fmol/L [35]. In photoelectrochemical immunoassays, PDA-coated zinc oxide nanorods improve charge separation and antibody attachment, achieving a detection limit of 10 pg/mL [32]. Environmental sensing benefits from PDA-coated zinc oxide nanorods with gold nanoparticles, enhancing photocurrent response for amyloid-beta detection, vital for early Alzheimer’s diagnosis [30]. Despite these advancements, there are gaps in optimizing PDA application protocols for various sensing platforms and understanding long-term stability and performance in practical settings. Addressing these gaps through comparative studies and long-term evaluations is crucial for fully achieving PDA’s potential in sensing, diagnostics, and analytical techniques.

Table 8 outlines concerns and limitations associated with polydopamine-based biosensing technologies, including the stability of enzyme activity, assay accuracy, the integration and long-term stability of biofunctional components, environmental and health impacts of nanoparticles, potential over-oxidation affecting biocompatibility, stability of polydopamine and immobilized nanoparticles, detachment of particles affecting reproducibility, understanding of polydopamine formation mechanisms, potential interference from complex sample matrices, and stability of bioconjugated interfaces.

## 10. Microfabrication and Nanoengineering

Polydopamine (PDA) has become a pivotal material in the field of microfabrication and nanoengineering, demonstrating significant utility in enhancing the functionality of microchips and nanostructured devices for complex biological and chemical analyses.

A notable example of PDA’s application is its use in conjunction with gold nanoparticles (PDA/Au NPs) to modify the surface of a PDMS microchip, aimed at enhancing electrophoretic performance for amino acid separation. This surface modification greatly improves the microchip’s hydrophilicity and biofouling resistance, leading to better wettability, stability, and reduced electroosmotic flow. These enhancements result in a fast and efficient separation process, illustrating an effective method for high-throughput biological analyses. This application not only improves the operational efficiency of the microchip but also extends its durability and functional lifespan in lab-on-a-chip systems [130].

Further extending its utility in capillary electrochromatography, PDA has been employed to immobilize bovine serum albumin (BSA) within PDMS microfluidic chips, creating a protein stationary phase for the enantioselective separation of amino acids. Here, the PDA coating not only enhances surface wettability and electroosmotic mobility but also facilitates the high-resolution separation of D- and L-tryptophan in remarkably short times, showcasing its potential to streamline protein-based separations in microfluidic devices [27].

Moreover, PDA has been utilized to tailor the inner surfaces of asymmetric nanopores, enhancing controlled ion transport in nanofluidic devices. This application leverages PDA’s versatility in chemical modification to improve the functionality of nanofluidic systems, making them more efficient for applications requiring precise molecular and ionic manipulation [131].

In nanopatterning, PDA has been applied to facilitate block copolymer designs on flexible substrates. This method utilizes sonication to achieve a polished, smooth surface finish essential for precise nanopatterning. Demonstrating PDA’s utility in creating durable and bend-resistant nanopatterns, this approach enhances the prospects for advanced roll-to-roll nanomanufacturing processes, which are crucial for the development of flexible electronics and wearable sensors [37].

Transitioning to biomedical applications, PDA-coated hemoglobin has been explored as an oxygen therapeutic, specifically the photocatalytic synthesis of PDA-coated annelid erythrocruorin. This process preserves the protein’s structure and functionality while enhancing its oxygen-binding capacity and antioxidant properties. Offering a promising alternative to traditional red blood cell transfusions, this approach could lead to the large-scale production of hemoglobin-based oxygen carriers with improved circulation and stability in vivo [132].

Additionally, PDA nanoparticles have been developed to mimic natural melanin for sunscreen applications, showcasing their effectiveness in protecting against UV-induced skin damage, enhancing skin barrier functions, reducing inflammation, and exhibiting potent antioxidant capabilities. Their biocompatibility and protective efficacy position these nanoparticles as superior alternatives to traditional sunscreen formulations, promising significant advancements in photoprotective skincare [133].

Further enhancing sunscreen functionality, PDA and lignin-based nanocapsules have been synthesized with exceptional bioadhesion properties. These nanocapsules not only provide enhanced retention on the skin but also deliver high sun protection factors (SPFs) with extended durability under UV exposure. The biocompatible and photostable features of these nanocapsules, combined with their high antioxidant capacity, ensure their effectiveness as long-acting sunscreens, demonstrating their potential in advanced sunscreen formulations [134].

Research on polydopamine (PDA) highlights its substantial benefits in enhancing microchips and nanostructured devices for complex biological and chemical analyses. PDA combined with gold nanoparticles (PDA/Au NPs) improves PDMS microchips by enhancing hydrophilicity and biofouling resistance, leading to better wettability, stability, and reduced electroosmotic flow, thus extending microchip operational efficiency [130]. In capillary electrochromatography, PDA immobilizes bovine serum albumin (BSA) within PDMS microfluidic chips, facilitating the high-resolution enantioselective separation of amino acids [27]. PDA also tailors the inner surfaces of asymmetric nanopores to enhance controlled ion transport in nanofluidic devices, improving molecular and ionic manipulation [131]. In nanopatterning, PDA enables smooth block copolymer designs on flexible substrates, crucial for advanced roll-to-roll nanomanufacturing processes [37]. Biomedical applications see PDA-coated hemoglobin as a potential oxygen therapeutic, enhancing oxygen-binding capacity and antioxidant properties [132]. PDA nanoparticles mimic melanin in sunscreen applications, providing effective UV protection and improving skin barrier functions [133]. Moreover, PDA and lignin-based nanocapsules deliver high sun protection factors with extended durability [134]. Despite these advancements, gaps remain in optimizing PDA applications, particularly in ensuring consistent performance across different conditions. Long-term studies are necessary to evaluate the stability and biocompatibility of PDA-modified materials, essential for fully achieving PDA’s potential in microfabrication and nanoengineering.

Table 9 presents concerns and limitations associated with diverse applications of polydopamine modification, including the potential leaching of nanoparticles affecting long-term use, limited information on stability and reproducibility of immobilized proteins, complexity in controlling uniformity of polydopamine deposition, scalability and durability of nanopatterns, understanding of long-term impacts on human health and the environment, bioaccumulation potential, optimization of processes for clinical settings, and scalability of production.

## 11. Environmental Applications

The field of environmental technology has been enriched by the innovative application of polydopamine-based surface modifications, which enhance water purification systems and pollutant monitoring capabilities. Among these innovations, the development of multifunctional chlorella microspheres coated with polydopamine stands out. These microspheres not only excel in removing heavy metals and oil from water but also possess magnetic and fluorescent properties, enabling both targeted movement and pollutant sensing. This combination of features provides a comprehensive solution to complex water pollution challenges, with the microspheres demonstrating exceptional pollutant removal efficiencies, magnetic guidance capabilities, and biosensing potential, highlighting their applicability in environmental cleanup [135].

Another notable advancement involves the creation of supramolecular hydrogels formed from polydopamine-coated clay and ferric ions, designed to remove organic pollutants like Rhodamine 6G from water. The aromatic moieties of polydopamine facilitate the efficient adsorption of these pollutants, while the hydrogels’ elastic response and self-healing properties enhance their practical utility in environmental applications [136].

The treatment of oily wastewater has also benefited from innovations involving polydopamine. Specifically, amino-functionalized multi-wall carbon nanotubes have been anchored onto polyether sulfone membranes using polydopamine. This modification significantly increases the membranes’ hydrophilicity and underwater oleophobicity, resulting in enhanced antifouling properties and high oil rejection rates. Such improvements ensure the effectiveness of these membranes in treating oily wastewater to meet stringent discharge standards [137].

Additionally, polydopamine has been employed in the extraction of hazardous compounds like polycyclic aromatic hydrocarbons, utilizing modified polytetrafluoroethylene stirrers in conjunction with graphene oxide and polydopamine. This method has exhibited exceptional efficiency and stability even in severe conditions, rendering it ideal for real-world environmental monitoring where precise detection and robust recovery rates are imperative [138].

The novel development of a jacket-free stir bar sorptive extraction method exemplifies the innovative use of polydopamine in environmental technology. By immobilizing cross-linked organic polymers on stainless steel wires, this method enhances the extraction efficiency of protoberberines from herb and rat plasma samples, showing excellent stability, reproducibility, and effectiveness in detecting low concentrations of these compounds with high recovery rates [139].

Expanding the scope of polydopamine’s applications, the stabilization of aluminum nanocrystals in aqueous solutions has been achieved using polydopamine coatings. This enhancement increases the surface area and stability of the nanocrystals, facilitating their use in surface-enhanced Raman spectroscopy for the sensitive detection of carcinogenic pollutants like benzo[a]pyrene, demonstrating sensitivity in the sub-parts per billion range [140].

In the enhancement in magnetic solid-phase extraction techniques, polydopamine-coated magnetic nanoparticles have been utilized to optimize the detection of estrogens in various water sources. These nanoparticles have shown superior performance compared to their carbon-coated counterparts, indicating stronger interactions with estrogen molecules and optimizing detection capabilities [141].

A mesoporous nano-absorbent combining polydopamine and graphene oxide has been developed to efficiently capture uranium(VI) from aqueous solutions. This system showcases rapid uranium capture and high adsorption capacity, making it suitable for a range of pH values and highlighting its potential in nuclear waste management and environmental decontamination [44].

For oil spill remediation, a bioinspired polydopamine/polyzwitterion coating has been applied to various substrates to enhance underwater anti-oil and anti-freezing properties. This one-step application method efficiently separates oil from water mixtures, demonstrating practical applications in environmental conservation and disaster response scenarios [142].

Exploring the utility of polydopamine in wildlife conservation, coatings applied to stainless steel tags for humpback whales have shown potential to reduce bacterial adhesion and infection risks. This application provides insights into enhancing the functional service lifetime of satellite telemetry tags used in marine biology research [143].

Moreover, the mildew-proofing capabilities of bamboo have been significantly improved through a polydopamine coating, which supports the growth of Ag and TiO_2_ nanoparticles on the surface. This treatment not only boosts bamboo’s resistance to fungal growth but also enhances its photocatalytic performance, demonstrating the versatility of polydopamine-modified materials in improving the durability and functionality of natural materials in environments prone to mildew [3].

Summing up, multifunctional chlorella microspheres coated with PDA exhibit superior heavy metal and oil removal capabilities, along with magnetic and fluorescent properties, making them highly effective for environmental cleanup [135]. Similarly, PDA-coated clay and ferric ion supramolecular hydrogels efficiently adsorb organic pollutants, utilizing PDA’s aromatic structures for pollutant removal [136]. In oily wastewater treatment, PDA-anchored amino-functionalized carbon nanotubes enhance membrane hydrophilicity and antifouling properties, crucial for stringent discharge standards [137]. Moreover, PDA-modified polytetrafluoroethylene stirrers with graphene oxide demonstrate high efficiency in extracting hazardous compounds, like polycyclic aromatic hydrocarbons, under harsh conditions [138]. Innovative jacket-free stir bar sorptive extraction using PDA-immobilized polymers improves protoberberine detection [139], and PDA-stabilized aluminum nanocrystals increase Raman spectroscopy sensitivity for carcinogen detection [140]. Additionally, PDA-coated magnetic nanoparticles enhance estrogen detection [141], and a PDA-graphene oxide nano-absorbent efficiently captures uranium(VI) [44]. For oil spill remediation, PDA/polyzwitterion coatings effectively separate oil from water [142], while PDA-coated whale tags reduce bacterial adhesion [143], and PDA-enhanced bamboo surfaces improve mildew resistance and photocatalytic performance [3]. Despite these advancements, gaps remain in optimizing PDA applications for consistent performance across varied environmental conditions, necessitating long-term studies to evaluate stability and efficacy.

Table 10 presents concerns and limitations associated with the environmental applications of polydopamine modification, including potential impacts on ecological balance, stability in aquatic environments, the toxicity of components, durability in real wastewater conditions, scalability of coating processes, leaching of materials, environmental impact from release of absorbed pollutants or breakdown of components, efficacy and safety in marine environments, and ecological impact of coatings over long periods.

## 12. Other Applications

A comprehensive review has meticulously analyzed the synthesis, properties, and broad applications of PDA, emphasizing its crucial functions in anchoring substances, promoting biomineralization, providing antifouling and antibacterial surfaces, facilitating drug and gene delivery, and advancing tissue engineering. The review highlights PDA’s intrinsic hydrophilicity, strong adhesive properties, and functional versatility, which together enhance the functionality of materials through innovative surface modifications [144].

Delving deeper into the interactions between PDA surfaces and biomolecules, one study utilized electrochemical impedance spectroscopy to explore how bovine serum albumin (BSA) binds to PDA-coated surfaces. This investigation shed light on the mechanisms of protein adhesion to PDA, showing different behaviors in nucleophilic versus non-nucleophilic buffers. The findings from this study are crucial, providing valuable insights into optimizing conditions for biomolecule immobilization on PDA surfaces, which is key for developing efficient bio-interactive interfaces [8].

Further extending the utility of PDA in creating specialized surfaces, PDA coatings have been functionalized through the aza-Michael reaction with acrylate/acrylamide molecules, including sulfobetaine acrylamide, to develop surfaces with significant antifouling and antimicrobial properties. These functionalized coatings have demonstrated excellent resistance to bacterial adhesion and have shown broad applicability across various substrates, underscoring their effectiveness in creating protective layers that are resistant to microbial colonization and fouling [145].

The surface, adhesion, and tribological behaviors of PDA films have been extensively investigated under both dry and wet conditions. These studies have revealed that PDA films exhibit distinctive behaviors influenced by hydration effects, which bear significant implications for their application as functional coatings and biocompatible interfaces. Particularly notable are the dynamic adhesion properties and the reduction in friction when hydrated, pointing to their potential use as effective lubrication coatings that could benefit a range of applications from industrial machinery to medical implants [4].

The modification of basalt fiber composites with PDA and graphene oxide has led to enhanced bonding and mechanical properties in basalt fiber/polyamide composites. This approach has successfully improved the wear resistance and load-bearing capacity of the composites, demonstrating the efficacy of PDA as a bridging agent that significantly bolsters adhesion and overall performance in composite materials [38].

In the realm of composite material enhancement, PDA coatings have been employed as a primer for silane grafting on bamboo fibers, which significantly improved their compatibility with poly(lactic acid) composites. This modification not only enhanced the mechanical properties and thermal stability of the composites but also presented a viable, environmentally friendly method for boosting the performance of natural fiber composites. The treated composites exhibited marked increases in flexural, tensile, and impact strength compared to their untreated counterparts, indicating a substantial enhancement in their structural integrity and durability [40].

Further innovations include the application of PDA and silica nanoparticles to coat carbon fibers, substantially enhancing the interfacial bonding and hydrothermal aging resistance of carbon fiber/polymer composites. This bioinspired approach has taken full advantage of PDA’s unique properties to improve both the mechanical strength and longevity of composites, indicating potential for broader applications in materials engineering [146].

The role of PDA in biomimetic mineralization processes was also showcased in a study that investigated its effectiveness in enhancing the mineralization of collagen type I with hydroxyapatite. This process formed c-axis-oriented platelets within the collagen matrix, demonstrating that PDA facilitates the nucleation and intrafibrillar mineralization of hydroxyapatite, providing valuable insights into strategies for developing biomimetic materials that mimic the properties of bone and dentin [147].

Moreover, PDA was utilized to modify the surface of paraffin microcapsules to enhance their adhesion within an epoxy matrix. This treatment effectively increased surface roughness, facilitating better mechanical interlocking and covalent bonding between the microcapsules and the matrix. The effectiveness of this surface modification was demonstrated through various spectroscopic and microscopic techniques, showing that the PDA coating significantly increased surface roughness and reactivity, leading to improved mechanical and chemical bonding properties [41].

PDA nanosheets were used to graft polymer brushes, creating surfaces with adjustable cell adhesion properties. These nanosheets provided a robust and detachable foundation for cell growth and non-fouling applications, demonstrating their versatility in biomedical applications. This innovative use of PDA highlighted its capability to promote cell growth and non-fouling behavior, dependent on the type of polymer brush used, showing potential for controlled cell adhesion applications in medical devices and tissue engineering [42].

One remarkable development involves the creation of colloidal polydopamine beads that serve as photothermally active supports for palladium nanocatalysts. This application capitalizes on PDA’s inherent photothermal properties to modulate catalytic reactions under light illumination. By harnessing PDA’s photothermal conversion capabilities, these beads enhance the efficiency of thermal catalytic processes, enabling precise control over reaction environments and outcomes. This approach opens new avenues for advancing catalysis technology by offering a method to dynamically control the kinetics of chemical reactions through light [43].

Furthermore, the synthesis, functionalization, and applications of polydopamine-coated magnetic iron oxide nanoparticles have been comprehensively reviewed. These nanoparticles have found extensive uses in biomedicine, sensing, and environmental remediation, benefiting significantly from PDA’s ability to enhance biocompatibility and introduce functional capabilities. The review highlighted the diverse applications of these nanoparticles, showcasing PDA’s versatility as a coating material that can be tailored to meet specific requirements in various sectors [50].

An innovative approach in surface engineering involves the development of an atmospheric plasma deposition process as an alternative to traditional dip-coating methods for applying polydopamine. This technique provides a faster, solvent-free method for depositing methacrylate layers containing catechol/quinone groups on metallic surfaces. Successfully applied in creating bioconjugation-ready biomedical surfaces, this method not only accelerates the coating process but also enhances antibiofilm properties, promoting early cell interaction and improving cell viability on medical implants and devices [148].

Additionally, a unified strategy for tailoring polydopamine and synthetic eumelanins has been presented, highlighting their similarities and distinct applications, ranging from UV protection to functioning as electronic–ionic semiconductors. This comprehensive study underscores the importance of understanding structure–property relationships in these materials, providing a framework for the future design of melanin-inspired materials for diverse technological applications. By exploring the unique properties of polydopamine and eumelanin, this research opens up potential for significant technological advancements in materials science [9].

To conclude, polydopamine (PDA) exhibits intrinsic properties such as hydrophilicity, strong adhesion, and functional versatility, enhancing surface modifications across various applications [144]. In biomolecular interactions, electrochemical impedance spectroscopy has revealed the binding behaviors of bovine serum albumin (BSA) to PDA-coated surfaces, differentiating between nucleophilic and non-nucleophilic buffers. This insight aids in optimizing biomolecule immobilization conditions on PDA surfaces, which is crucial for developing efficient bio-interactive interfaces [8]. Functionalized PDA coatings, particularly through the aza-Michael reaction, have demonstrated exceptional antifouling and antimicrobial properties, extending their applicability across various substrates [145]. Studies on PDA films’ adhesion and tribological behaviors indicate dynamic adhesion properties and reduced friction when hydrated, suggesting their potential as lubrication coatings for industrial and medical applications [4]. PDA-modified composites, such as those with graphene oxide or silane-grafted bamboo fibers, exhibit enhanced mechanical properties and stability, highlighting environmentally friendly methods to improve natural fiber composites [38,40]. PDA coatings on carbon fibers and their role in biomimetic mineralization processes further underscore PDA’s versatility in enhancing material performance and mimicking biological properties [146,147]. Despite significant advancements, there remain gaps in optimizing PDA applications for consistent performance across different environments, necessitating comprehensive long-term studies to evaluate their stability, efficacy, and scalability [9,148].

Table 11 summarizes concerns and limitations associated with a wide range of polydopamine modification applications, including the durability of antimicrobial properties, competitive protein binding, environmental and health impacts of residues, dehydration-induced cracking of coatings, stability of catalyst systems, variability in mineralization efficiency, environmental impact of nanoparticles, long-term stability and biocompatibility of deposited materials, optimization of material performance, adjustments affecting composite performance, challenges in layer integrity, and optimization of coating thickness and uniformity.

## 13. Key Factors Determining the Polydopamine Modification for Various Applications

The selection of polydopamine (PDA) modification methodologies is a multifaceted process influenced by a variety of factors tailored to the intended application. For bone tissue engineering, critical considerations include the desired mechanical properties, biocompatibility, and osteogenic potential of the materials. The type of substrate to be modified, such as calcium silicate [12], hydroxyapatite composites [13], or titanium alloys [14], dictates specific approaches to optimize surface properties for enhanced integration and performance. Cellular interactions, particularly the proliferation and differentiation of specific stem cells like Wharton’s jelly mesenchymal stem cells (WJMSCs) [12] or rat mesenchymal stem cells (rMSCs) [15], guide the selection of PDA modifications to support these biological processes effectively.

The intended application is a primary driver in choosing PDA modification methodologies. Enhancing general osteointegration [14] requires modifications that improve the adhesion and growth of bone cells on implant surfaces, while promoting subchondral bone repair [18] necessitates strategies for rapid and effective bone regeneration. For instance, bone morphogenetic protein-2 (BMP-2) peptides on PDA-coated scaffolds [15] are selected for applications needing sustained release of osteogenic differentiation cues to stimulate bone growth.

Clinical requirements and the desired biological responses significantly influence the choice of modification methods for implant enhancements. In bone tissue applications, methodologies that enhance osteogenesis and reduce bone resorption, such as PDA combined with tannic acid or epsilon-polylysine, are prioritized for their efficacy in promoting bone cell activity and integration [16,21]. Implants in areas prone to infection benefit from methodologies incorporating antibacterial agents like silver or epsilon-polylysine, which prevent post-surgical infections [20,21]. For improved soft tissue integration, the PDA-mediated covalent bonding of collagen ensures enhanced cellular adhesion and biocompatibility, essential for successful integration with soft tissues [6].

Environmental considerations play a significant role in selecting PDA modification methodologies for non-medical applications. The nature of the target pollutant, whether heavy metals, organic pollutants, or oils, determines the strategy for effective removal. Multifunctional chlorella microspheres are chosen for their broad-spectrum pollutant removal capabilities [135], while specific aromatic moieties in hydrogels target organic pollutants [136]. Environmental conditions and application contexts, such as the stability and performance of modified materials under varying pH levels and in the presence of other contaminants, are crucial factors. An example is the development of nano-absorbents for uranium capture, which must remain stable and effective under diverse environmental conditions [44].

Practical factors, including ease of material synthesis, cost-effectiveness, reusability, and high recovery rates, are essential in determining the most suitable modification method. The development of PDA-based coatings for bamboo to enhance mildew resistance and photocatalytic performance exemplifies how these factors are balanced to achieve durable and multifunctional environmental solutions [3]. In energy and electronics applications, considerations include the structural integrity and functional efficiency of nanocomposites, necessitating precise control over integration and morphology [97,103]. The ability to achieve multifunctional properties, such as enhanced hydrophilicity or specific binding capabilities, often guides the choice of methodology in applications ranging from environmental engineering to tissue engineering [45,99,101].

Moreover, the integration of PDA in biomedical applications must address biocompatibility and bioactivity to enhance scaffold surfaces for improved cell adhesion and differentiation [73,107]. Mechanical properties such as rigidity and stickiness are vital in applications requiring structural support, such as integrating PDA into 3D electrospun PCL sponges for tissue engineering and drug delivery [39]. Specific functional attributes, such as anti-inflammatory effects in pelvic organ prolapse repair [36] or enhanced hemocompatibility in vascular devices [111], dictate the choice of PDA modification techniques. Stability and durability are critical, particularly in dynamic environments like neural recording applications, requiring mechanically stable platinum black microelectrodes [34].

The compatibility of PDA modifications with the biological environment, along with specific biomedical application requirements, guides the selection of appropriate methodologies to optimize the performance and efficacy of tissue engineering and cell culture technologies. For instance, in neurological applications, enhancing blood–brain barrier (BBB) penetration and brain tissue uptake necessitates glucose modifications on PDA coatings [123]. In dermatology, drug loading capacity and penetration depth are crucial, leading to the preference for mesoporous nanoparticles with PDA coatings [124]. For vascular applications, promoting endothelial cell proliferation while minimizing smooth muscle cell growth guides the choice of immobilizing gene complexes on PDA-coated substrates [93]. Optimizing nanoparticle synthesis for drug delivery involves factors like size, elasticity, and stability, with PEGylation enhancing these properties in PDA nanoparticles [46]. For gene therapy applications, such as siRNA delivery, enhancing cellular uptake and gene silencing efficacy is key, with PDA coatings facilitating effective siRNA attachment and transmembrane delivery [125].

The selection of PDA modification methodologies is a complex process influenced by a multitude of factors tailored to the specific requirements of the intended application. Whether for biomedical, environmental, or industrial purposes, considering aspects such as biocompatibility, mechanical properties, environmental impact, and practical feasibility ensures that PDA-based materials perform optimally in their respective contexts. By carefully aligning these methodologies with the unique needs of each application, researchers can enhance the efficacy and functionality of PDA-modified materials, achieving the desired outcomes across various fields.

Additionally, recent studies further illustrate the broad utility of PDA and its analogues in diverse applications. For instance, the vast use of implants in orthopedic surgeries has driven a tremendous demand for surface modifications to enhance implant function and reduce surgery failure. PDA, with its ability to adhere to various substrates and immobilize biomolecules and metal ions, has proven beneficial in modulating cellular responses such as spreading, migration, proliferation, and differentiation. This enhances the function and osseointegration of orthopedic implants while providing antimicrobial properties [60]. Polydopamine analogues (PDANAs) expand the capabilities of PDA by incorporating additional functional groups, achieved through post-functionalization, oxidative polymerization, or copolymerization with other monomers or reactive polymers. This broadens the range of properties and applications for these materials, highlighting the future potential in various fields [61]. In membrane technology, functional groups on support membranes are crucial for high-performance membrane adsorbers (MAs). Surface modifications, such as alkali treatment, oxidation, and PDA deposition, improve adsorption performance. For instance, PDA deposition on membranes enhances protein adsorption capacity, offering potential advancements in this area [62]. PDA hydrogels also demonstrate significant promise, with their abundant functional groups allowing for various covalent and non-covalent interactions with polymers and transition metal ions. These interactions impart diverse functions to hydrogels, such as adhesion, photothermal effects, ultraviolet protection, antioxidant ability, and antibacterial properties, enabling their application across biomedical, environmental, energy, and electronic fields [63]

## 14. Testing and Assessment of the Polydopamine Products

Conducting a comprehensive array of tests is crucial to fully understand and harness polydopamine (PDA)’s potential across various applications.

### 14.1. Material Characterization and Surface Analysis

Structural and Surface Characterization: To begin with, researchers employ Scanning Electron Microscopy (SEM) to examine the morphology of PDA coatings and structures [12,14,19,25,36,39]. Field Emission Scanning Electron Microscopy (FESEM) and Transmission Electron Microscopy (TEM) can provide more detailed surface and internal structural images [14]. For elemental and chemical state analyses, X-ray Photoelectron Spectroscopy (XPS) is essential [3,12,23,24,25,68,75,79,100,105,109,123]. Additionally, Atomic Force Microscopy (AFM) should be used to assess the surface topography and roughness at the nanoscale [8,22,23,36,41,46,134].

### 14.2. Biomedical Applications and Biocompatibility Testing

Cell-Based Assays: It is critical to evaluate the biocompatibility of PDA through various cell-based assays, which assess cell adhesion, proliferation, and differentiation on PDA-coated surfaces [13,17,18,21,35,36,73,108,109]. These tests are foundational in applications such as tissue engineering and regenerative medicine.

In Vivo Testing: To extend laboratory findings to practical applications, in vivo tests are necessary. These may include bone regeneration models in rats or rabbits to evaluate the osteogenic potential of PDA-modified materials [15,35,73] and studies on implant integration within biological systems [68,108,111].

### 14.3. Environmental Applications and Stability Assessments

Pollutant Capture and Sensitivity Assessments: For PDA applications in environmental technology, tests for pollutant capture efficacy and sensitivity are vital [140]. Additionally, oil/water separation tests help in determining the efficiency and reliability of PDA in water purification processes [137,142].

Antimicrobial Tests: To explore PDA’s antimicrobial properties, researchers should conduct microbial adhesion and viability assays to assess the antimicrobial efficacy of PDA surfaces [145,148].

### 14.4. Enhancement in Biosensing Capabilities

Biosensor Development: For the development of sensitive and selective biosensors, techniques such as electrochemiluminescence (ECL) [35], photoelectrochemical method (PEC) [32], and surface-enhanced Raman scattering (SERS) [29,127] should be utilized. These methods help in enhancing the detection capabilities of biosensors for clinical and environmental monitoring.

### 14.5. Advanced Functional and Material Performance Evaluations

Mechanical and Electrochemical Testing: Mechanical integrity tests, such as compression and bending tests, are important for understanding the durability and mechanical properties of PDA coatings under stress [18]. Electrochemical tests, including cyclic voltammetry and electrochemical impedance spectroscopy, are crucial for applications in energy storage and conversion [8,99].

By incorporating these diverse testing methodologies, researchers can effectively characterize and optimize PDA for a wide range of applications. Each test contributes significantly to understanding the complex interactions and properties of PDA, ultimately facilitating its successful integration into innovative material solutions and medical advancements.

## 15. Limitations in Polydopamine-Related Research

Complexity in Synthesis and Functionalization: The synthesis and functionalization of polydopamine (PDA) are complex and challenging to scale up, which affect the consistency in coating quality and functional performance across larger batches. This complexity poses a barrier to large-scale production and achieving consistent quality [15,30,36,57,73,74,75,133].

Incomplete Understanding of Mechanisms and Long-Term Effects: Despite extensive research, the exact mechanisms of interaction between polydopamine and biological tissues remain poorly understood. Additionally, the long-term stability and potential degradation products of PDA under physiological and various environmental conditions are not fully understood, impacting the predictability, optimization, safety, and effectiveness of polydopamine-based materials [1,16,17,19,51,73,102,108,135,136].

Variability in Biocompatibility and Performance: Although generally biocompatible, different modifications and applications of PDA can alter its interaction with biological systems, necessitating extensive testing for each new application. Achieving consistent quality and functionality across different substrates and environmental conditions remains challenging, affecting the reliability and predictability of PDA-enhanced materials [5,32,57,107,109].

Complexity in Functional Integration: Integrating polydopamine into complex biomedical systems such as nerve regeneration devices or vascular stents requires precise control and a deep understanding of interactions at the biomaterial interface, which is still under extensive research [35,111].

Potential for Incomplete Integration in Biomedical Systems: While polydopamine shows promise in biomedical applications, achieving complete integration and functionality in complex biological systems remains a challenge, necessitating further research to optimize interactions at the biological interface [93,123].

## 16. Future Directions in Polydopamine-Related Research

Improving Synthesis and Application Techniques: Focus research on developing efficient, scalable methods for synthesizing and applying polydopamine (PDA) coatings that ensure high quality and functional integrity, necessary for both commercial and medical scalability [15,36,48,73,74,75,79,106]

Deeper Mechanistic Studies and Comprehensive Evaluations: Conduct further studies to better understand the interactions of PDA with various biological systems and its long-term stability, degradation, and biocompatibility across different environmental and physiological conditions. This knowledge is critical for improving the design of bioactive materials and ensuring the safety and effectiveness of PDA in long-term applications [1,16,17,19,44,51,57,73,136].

Expanded Applications in Biomedicine and Beyond: Explore broader applications of PDA in biomedicine, including wound healing, neural interfaces, and drug delivery systems. Additionally, leverage PDA’s unique properties in non-medical fields, such as environmental engineering, sensors, and energy storage, to address environmental challenges and develop new industrial solutions [92,93,96,97,103,111,135,137].

Advanced Biomedical Engineering Applications: Innovate in areas like targeted drug delivery, advanced imaging techniques, and next-generation medical devices. Enhance the integration of PDA in developing advanced applications like vascular grafts or neural interfaces, where its multifunctional properties can control biological interactions and improve medical outcomes [57,75,93,123,124,133].

Development of Advanced Sensing Technologies: Continue innovation in biosensing technologies using PDA to create more sensitive and selective detection systems for early disease diagnosis and environmental monitoring. This effort could lead to improved healthcare diagnostics and environmental safety [30,32,73].

Enhancement in Biomedical and Environmental Applications: Further research into enhancing drug delivery systems, developing effective medical implants, and exploring PDA’s potential in regenerative medicine and environmental remediation. This includes expanding its use in pollutant detection, water treatment, and sustainable material production, ensuring its effectiveness in long-term applications [38,40,50,93,123,136,146].

## 17. Conclusions

Polydopamine (PDA) has emerged as a versatile and potent material in both the biomedical and environmental fields, showing significant promise in enhancing biocompatibility, osteointegration, and antibacterial properties, alongside robust applications in cancer treatment and water remediation. Despite these achievements, challenges such as complex synthesis processes, incomplete mechanistic understanding, and variability in biocompatibility underscore the need for further research. Future directions should concentrate on simplifying synthesis methods, expanding applications beyond traditional boundaries, and conducting comprehensive evaluations to better understand and optimize PDA’s interactions with biological systems. Addressing these limitations will not only enhance the functional integrity and scalability of PDA-based materials but also broaden their impact across diverse scientific and industrial domains.

## Figures and Tables

**Table 1:** Polydopamine modifications and limitations in coatings and surface modifications.

Modification of Polydopamine	Concerns and Limitations	Ref.
Enhanced antibacterial performance of Foley catheters through amino acid-conjugated polydopamine surfaces.	Potential long-term stability and effectiveness of the amino acid conjugation on medical device surfaces.	[22]
Dual-function polydopamine coatings provide antimicrobial and antifouling properties for medical devices.	Durability of the coating under physiological conditions and potential copper ion toxicity.	[24]
Antibacterial calcium phosphates triggered by polydopamine enhance osteoblast activity and inhibit pathogenic bacteria.	Potential cytotoxicity from silver nanoparticle leaching and long-term environmental impact.	[25]
Polydopamine coating of diatom microalgae increases their environmental resistance, offering applications in biotechnology.	Potential impact of polydopamine on the natural ecological functions and interactions of coated diatoms.	[72]
Antifouling and antimicrobial polymer membranes developed using polydopamine for improved biocompatibility.	Stability and robustness of the hydrogen-bonded poly(N-vinyl pyrrolidone) layers in various applications.	[76]
Polydopamine coatings on nitric oxide releasing polymers decrease bacterial infections and thrombus formation.	Durability and effectiveness of the coatings under long-term physiological conditions.	[77]
Polydopamine coatings promote the nucleation and growth of biomimetic minerals, enhancing biomaterial integration.	Effects of PDA coating on long-term mineralization and interfacial stability with tissues.	[78]
Preparation of stable superhydrophobic coatings on wood via polydopamine enhances durability and environmental resistance.	Feasibility and environmental impact of scaling up the production of superhydrophobic wood coatings.	[79]
Enhanced mechanical properties of polydopamine coatings through thermal annealing without losing functionality.	Thermal stability and potential degradation of polydopamine properties over time or in vivo conditions.	[80]
Polydopamine layer as a versatile functionalization protocol for silicon-based photonic biosensors.	Consistency and stability of biofunctionalization across different biomarkers and environmental conditions.	[81]

**Table 2:** Polydopamine modifications and limitations in bone tissue engineering.

Modification of Polydopamine	Concerns and Limitations	Ref.
Polydopamine facilitates the biomineralization of hydroxyapatite, improving implant integration and cellular interactions.	Potential for inflammatory responses or immune reactions to foreign materials.	[1]
Enhanced cell adhesion and proliferation due to PDA-coated calcium silicate surfaces.	Long-term effects and stability of polydopamine coatings in vivo remain unclear.	[12]
Polydopamine incorporation into biomaterials increases mechanical strength and promotes bone regeneration effectively.	The exact dose and degradation behavior of polydopamine in biological environments need further study.	[13]
Polydopamine-assisted hydroxyapatite coatings on titanium scaffolds significantly improve osteointegration and bone regeneration.	Concerns about the wear debris from coated titanium and its biological effects.	[14]
Sustained delivery of BMP-2-derived peptides from polydopamine-coated scaffolds enhances bone induction effectively.	Potential issues with peptide release kinetics and control over long-term functionality.	[15]
Polydopamine-regulated hydroxyapatite microspheres improve mechanical properties and osteogenic activity in biomimetic scaffolds.	Long-term biocompatibility and mechanical stability of the scaffold under physiological conditions need further investigation.	[18]
Enhanced osteogenic differentiation and bone regeneration using polydopamine-coated biomimetic scaffolds loaded with exosomes.	Potential risks of exosome variability and immunogenicity could affect scaffold performance.	[19]

**Table 3:** Polydopamine modifications and limitations in implant enhancement.

Modification of Polydopamine	Concerns and Limitations	Ref.
Polydopamine-mediated covalent functionalization of collagen on titanium enhances biocompatibility with soft tissues.	The durability and long-term effects of collagen integration on tissue response are not fully understood.	[6]
Reduction in osteoclast activity by polydopamine and polyphenol tannic acid coatings, which could improve osseointegration at the bone–implant interface.	Specific long-term effects of tannic acid on human cells and tissues remain to be fully understood.	[16]
Mussel-inspired polydopamine coatings enhance osteogenic differentiation and osseointegration across various implant materials.	Consistency and durability of the polydopamine coating in different environmental conditions and its effect over time.	[17]
Polydopamine-induced Ag/CaP coatings on titanium nanotubes offer antibacterial and osteointegration properties.	Potential cytotoxicity and long-term stability of silver nanoparticles need to be carefully evaluated.	[20]
Polydopamine-assisted layer-by-layer coatings enhance antibacterial and osteogenic properties of titanium implants.	Balancing long-term antibacterial effectiveness with tissue compatibility and potential toxicity of coated materials.	[21]

**Table 4:** Polydopamine modifications and limitations in cancer treatment and imaging.

Modification of Polydopamine	Concerns and Limitations	Ref.
Polydopamine-modified nanocarriers provide enhanced tumor imaging and chemo-photothermal therapy.	Concerns about the systemic toxicity and precise targeting of these nanocarriers in human therapy.	[74]
Polydopamine surface engineering on nanoparticles can inhibit exocytosis and enhance cancer therapy by impairing lysosomes.	The potential for unintended cellular effects and resistance due to lysosomal impairment needs further investigation.	[92]
Polydopamine-modified iron-immobilized MnPS(3) nanosheets enable multimodal imaging and synergistic cancer therapy.	Efficient excretion and safety of the materials in clinical settings remain to be fully assessed.	[93]
Polydopamine core–shell nanostructures enable precise combination therapy with controlled drug release for breast cancer.	Potential toxicity and long-term biodegradation of polydopamine nanoparticles in vivo.	[94]
Amyloid protein-biofunctionalized polydopamine nanoparticles show minimal plasma protein fouling and efficient photothermal therapy.	Long-term impact and safety of amyloid proteins in clinical applications.	[95]
Polydopamine-based nanoplatform allows the multilayer imaging of cancer biomarkers from extracellular vesicles.	Specificity and accuracy in complex biological samples and the long-term stability of the nanoplatform.	[96]

**Table 5:** Polydopamine modifications and limitations in nanotechnology applications.

Modification of Polydopamine	Concerns and Limitations	Ref.
Polydopamine/Ag nanocomposite particles exhibit enhanced antibacterial activities with good cytocompatibility.	Potential environmental and health impacts due to silver leaching.	[5]
Bioinspired polydopamine used for medical applications, such as tumor ablation and drug delivery.	Potential immune reactions or cytotoxicity due to chronic exposure to polydopamine-based materials.	[7]
Mussel-inspired functionalization of graphene with polydopamine and silver nanoparticles provides strong antibacterial properties.	Concerns about silver nanoparticle leaching and resistance development in target bacterial populations.	[23]
Green strategy for the surface engineering of magnetic nanoparticles (MNPs) integrates PET-ATRP with polydopamine chemistry.	Potential long-term environmental impact and biocompatibility of modified MNPs.	[45]
Magnetic Fe_3_O_4_-polydopamine hybrid hollow microspheres show intrinsic peroxidase-like activity and magnetic separability.	Stability and potential leaching of magnetic nanoparticles in biological or environmental applications.	[48]
Synthesis of mesoporous silica/polymer hybrids for specific applications like lithium isotope separation.	Complexity in controlling the uniformity of polymer growth and functionality in hybrid materials.	[49]
Polydopamine-coated nanoparticles demonstrate ultrastable coatings and improved biocompatibility in vivo.	Long-term in vivo stability and potential accumulation of nanoparticles in the liver and spleen.	[51]
Polydopamine-coated gold nanorods functionalized with antibodies allow targeted cancer cell imaging and photothermal therapy.	Potential for off-target effects and immune responses to nanorod constructs in vivo.	[57]
Polydopamine melanin-mimetic nanoparticles allow for intracellular imaging with size control and fluorescence labeling.	Reproducibility of particle synthesis and control over functional loading.	[75]
Integration of inorganic nanostructures with polydopamine-derived carbon enhances electrical and mechanical properties.	Long-term environmental stability and potential toxicity of nanostructures need further investigation.	[97]
Polydopamine-modified nanodiamonds as a platform for bio-functionalization and metal nanoparticle reduction.	Risk of nanoparticle aggregation or instability in biological or environmental applications.	[98]
Electropolymerization of polydopamine for electrode-supported insulating mesoporous films for ion transport control.	Potential issues with scaling and replicating electropolymerization processes consistently across applications.	[99]
Development of a versatile polydopamine-like platform for hydrophobic and potentially bioactive surface coatings.	Long-term stability and performance of the coatings in various environmental conditions.	[100]
Polydopamine-poly(ethylene oxide) surfaces modified with peptides enhance cell adhesion and growth in tissue engineering.	Optimization of peptide density and composition for improved functional outcomes is needed.	[101]
Dual-functional polydopamine layers on nanofiltration membranes enhance antifouling properties and flux.	Possible challenges in scaling up the fabrication process for commercial applications.	[102]
Polydopamine acts as an effective electron gate for artificial photosynthesis, enhancing photochemical water oxidation efficiency.	Stability and efficiency of the polydopamine layer under prolonged exposure to environmental conditions.	[103]
Polydopamine adhesion facilitates the functionalization of liquid-crystalline elastomers with conductive copper.	Reduced conductivity due to mechanical stress and cracking during actuation.	[104]
Improved immobilization of biomolecules to quinone-rich polydopamine for surface functionalization.	Durability and effectiveness of the biomolecule immobilization under physiological conditions.	[105]
Polydopamine enables controlled synthesis of various nanomaterials for diverse applications.	Long-term stability and reproducibility of the synthesized nanomaterials.	[106]

**Table 6:** Polydopamine modifications and limitations in tissue engineering and cell culture.

Modification of Polydopamine	Concerns and Limitations	Ref.
Electrochemical layer-by-layer fabrication of mechanically stable platinum black microelectrodes using polydopamine for neural signal recording.	Durability and long-term performance of the microelectrodes in a biological environment.	[34]
Polydopamine-decorated PLCL conduit induces synergetic effects of electrical stimulation and topological morphology for peripheral nerve regeneration.	Evaluating long-term effects and functional recovery consistency in larger clinical trials.	[35]
Preparation of a small intestinal submucosa-modified polypropylene hybrid mesh via polydopamine coating enhances biocompatibility and reduces inflammation for pelvic reconstruction.	Potential long-term compatibility and the mechanical integrity of the hybrid mesh in the human body.	[36]
Polydopamine inter-fiber networks increase the rigidity and functionality of 3D electrospun fibrous sponges.	Potential challenges with scale-up production and maintaining consistent properties across batches.	[39]
Polydopamine nanospheres facilitate the biomimetic mineralization of hydroxyapatite, enhancing biocompatibility and bioactivity for tissue engineering.	Long-term in vivo stability and performance of the hydroxyapatite synthesized on PDA nanospheres.	[47]
Hierarchically patterned polydopamine-containing membranes promote periodontal tissue regeneration with enhanced osteogenic differentiation.	Ensuring consistent properties and long-term stability of the biomimetic membranes in clinical settings.	[73]
Enhanced adhesion of preosteoblasts in 3D PCL scaffolds by polydopamine coating improves scaffold functionality for tissue engineering.	Possible variability in coating effectiveness across different scaffold batches or scale-up production.	[107]
Polydopamine-induced hydroxyapatite coating on implants improves osteogenesis and integration with bone tissue.	Potential risks associated with polydopamine degradation products and their effects on surrounding tissues.	[68]
Polydopamine-coated paper-stack nanofibrous membranes enhance adipose stem cells’ adhesion and osteogenic differentiation.	Long-term viability and functionality of cells in 3D stacked scaffolds for tissue engineering.	[108]
Polydopamine-coated PDMS enhances mesenchymal stem cell adhesion and multipotency for long-term culture.	Long-term stability of the dopamine coating under physiological conditions.	[109]
Polydopamine-mediated surface modification enhances stem cell differentiation and proliferation on scaffold materials.	Consistency and reproducibility of polydopamine coatings in large-scale applications.	[110]
Mussel-inspired polydopamine coating enhances endothelial cell function and reduces smooth muscle cell proliferation on vascular devices.	Long-term effects of polydopamine coatings on cell behavior and hemocompatibility in clinical settings.	[111]

**Table 7:** Polydopamine modifications and limitations in controlled delivery.

Modification of Polydopamine	Concerns and Limitations	Ref.
Bioinspired polydopamine nanoparticles offer controlled drug delivery with enhanced PEGylation for improved physiological stability.	Challenges in achieving consistent nanoparticle properties and performance across batches.	[46]
Virus-inspired glucose and polydopamine coating enhances brain drug delivery efficiency across the blood–brain barrier.	Potential toxicity or side effects of nanoparticles used in the brain.	[123]
Polydopamine-based nanoparticles show promise in skin drug delivery, enhancing penetration and protection against photodegradation.	Long-term effects and possible toxicity of chronic exposure to nanoparticles in the human body.	[124]
High-density SiNWs treated with polydopamine enhance gene-silencing efficiency by promoting cell membrane perturbation and improving siRNA delivery.	Potential cytotoxicity from high aspect ratio nanomaterials and their long-term effects on cellular health and behavior.	[125]
Polydopamine coating facilitates substrate-mediated gene delivery, enhancing endothelial cell competitiveness for vascular implants.	Risks associated with gene therapy, including potential off-target effects and immune responses.	[126]

**Table 8:** Polydopamine modifications and limitations in sensing, diagnostics, and analytical techniques.

Modification of Polydopamine	Concerns and Limitations	Ref.
Polydopamine coatings offer simple and robust modifications for capillary electrophoresis, improving analytical performance.	Lack of detailed understanding of PDA formation mechanisms may limit optimization and reproducibility of coating processes.	[2]
Polydopamine-assisted immobilization of zeolitic imidazolate framework-8 enhances capillary electrochromatography with improved phase ratio and interaction with analytes.	Noncovalent attachment could lead to detachment of particles under high flow rates or pressures, affecting reproducibility.	[26]
Improved antibody immobilization on polydopamine thin films enhances sensitivity and capacity in surface plasmon resonance immunoassay.	Potential over-oxidation could lead to decreased biocompatibility and structural integrity of the polydopamine film.	[28]
Bioinspired polydopamine surfaces can efficiently immobilize biomolecules and nanoparticles, enhancing sensitivity in multiple biosensing applications.	Stability of the polydopamine and potential leaching of immobilized nanoparticles could affect long-term performance.	[29]
ZnO@polydopamine/Au nanocomposites show enhanced photocurrent response for sensitive and reliable PEC immunoassay of amyloid-beta protein, potentially aiding early Alzheimer’s detection.	Concerns regarding the environmental and health impacts of nano-waste, including potential toxicity of nanoparticles if released.	[30]
Bioinspired PDA improves biosensor performance by facilitating active AuNPs anchoring and graphene oxide reduction.	Stability of the bioconjugated interface under real-world conditions.	[31]
Bioinspired polydopamine thin film enhances charge separation and antibody attachment, improving the reliability of label-free photoelectrochemical immunoassays.	Integration and long-term stability of the biofunctional components on the PEC interface may affect sensor performance.	[32]
Polydopamine-imprinted polymer on metal–organic frameworks allows the highly selective fluorescence detection of metronidazole, enhancing analytical specificity.	Potential interference from complex sample matrices in practical applications could affect detection accuracy.	[33]
Effective enrichment and duplex SERS detection using TiO_2_ nanorods@PDA/Ag enhances food safety monitoring.	Potential environmental impact and safety of nanoparticles.	[127]
Recombinant FPO immobilized on polydopamine-coated membranes allows the efficient and stable colorimetric determination of HbA1c, enhancing diabetes management.	Stability of enzyme activity over longer periods and potential variability in assay results due to external factors.	[128]
Polydopamine-functionalized nanoparticles enable highly sensitive electrochemiluminescent thrombin assays with good stability and selectivity, suitable for clinical applications.	Possible interference or cross-reactivity in complex biological samples that could affect the assay’s accuracy.	[129]

**Table 9:** Polydopamine modifications and limitations in microfabrication and nanoengineering.

Modification of Polydopamine	Concerns and Limitations	Ref.
Facilitates protein immobilization for chip-based open tubular capillary electrochromatography enantioseparation.	Limited information on the long-term stability and reproducibility of the immobilized proteins.	[27]
Enables block copolymer nanopatterning on flexible substrates with an ultrasmooth surface.	Concerns about the scalability and durability of the nanopatterns in real-world applications.	[37]
Enhanced electrophoresis separation of amino acids through a polydopamine/gold nanoparticle hybrid coating.	Potential leaching of nanoparticles affects long-term use.	[130]
Tailors the functional properties of solid-state nanopores for controlled ion transport.	Complexity in controlling the uniformity of polydopamine deposition inside nanopores.	[131]
Photocatalytic synthesis preserves the structure of high-molecular-weight hemoglobin for use in transfusion medicine.	Optimization of the photocatalytic process for clinical settings and scalability of the production.	[132]
PDA nanoparticles enhance photoprotection against UV-induced skin damage, with antioxidant and anti-inflammatory properties.	Potential long-term impacts of nanoparticles on human health and the environment are not fully understood.	[133]
Bioadhesive lignin-PDA nanocapsules improve sunscreen performance with high SPF values and good water resistance.	Bioaccumulation potential of nanomaterials and their environmental impact remain of concern.	[134]

**Table 10:** Polydopamine modifications and limitations in environmental applications.

Modification of Polydopamine	Concerns and Limitations	Ref.
Facile in situ growth of Ag/TiO_2_ on polydopamine-modified bamboo improves mildew proofing with a good photocatalytic performance.	Environmental impact of Ag and TiO_2_ nanoparticles if released into the environment.	[3]
Polydopamine–graphene oxide/hydroxyapatite composites effectively remove uranium from aqueous solutions.	Possible leaching of uranium or other toxic elements during use or disposal of the composites.	[44]
Polydopamine-mediated functionalization of chlorella microspheres enhances their utility in environmental applications like heavy metal and oil removal.	Potential impact on the natural ecological balance and functionality of modified chlorella in ecosystems.	[135]
Polydopamine-modified clay and ferric ions create a framework for pollutant-absorbing supramolecular hydrogels.	Potential environmental impact from the release of absorbed pollutants or breakdown of hydrogel components.	[136]
Bioinspired polydopamine coating enhances the hydrophilicity and oleophobicity of PES membranes for oily wastewater treatment.	Long-term durability of the coatings and efficiency in real wastewater conditions.	[137]
Polydopamine-modified polytetrafluoroethylene stirrers improve the extraction efficiency of polycyclic aromatic hydrocarbons.	Compatibility and efficiency of graphene oxide coatings in varied analytical applications.	[138]
Polydopamine-functionalized stir bars provide the efficient extraction of bioactive compounds and stability under harsh conditions.	Scalability of the coating process and potential leaching of materials during extraction.	[139]
Polydopamine-stabilized aluminum nanocrystals offer aqueous stability and enable the detection of polycyclic aromatic hydrocarbons.	Stability of the polymer in long-term aquatic environments and potential toxicity of nanocrystal components.	[140]
Bioinspired polydopamine/polyzwitterion coatings offer underwater anti-oil and anti-freezing properties.	Durability and ecological impact of the coatings in natural water bodies, especially over long periods.	[142]
Polydopamine coating on stainless steel satellite telemetry tags potentially reduces bacterial adhesion, improving tag lifespan for whale monitoring.	Efficacy and environmental safety of hydrogen peroxide release in marine environments.	[143]

**Table 11:** Polydopamine modifications and limitations in other applications.

Modification of Polydopamine	Concerns and Limitations	Ref.
Polydopamine thin films improve lubrication and adhesion under wet conditions, potentially for biocompatible coatings.	Dehydration can lead to cracking and reduced durability of PDA coatings on softer substrates.	[4]
Enhanced protein binding efficiency on biomimetic polydopamine surfaces under specific conditions.	Competitive binding may limit the efficiency of protein immobilization under certain conditions.	[8]
Integration of melanin-inspired materials for advanced functional applications in energy and medicine.	Need for further understanding of structure–property relationships to optimize material performance.	[9]
Improved mechanical and tribological properties of basalt fiber composites through interfacial modification.	Potential environmental and health impacts of graphene oxide and polydopamine residues.	[38]
Improved interface compatibility and performance of bamboo fiber in PLA composites through PDA and silane grafting.	Concerns about the long-term environmental impact of silane compounds in composite materials.	[40]
Increased adhesion between microcapsules and epoxy matrix in thermal management applications.	Adjustments in deposition parameters could affect PDA layer properties and composite performance.	[41]
Direct photografting of polymer brushes on PDA nanosheets enables controlled cell adhesion and nonfouling behavior.	Potential challenges in the detachment process from substrates could affect layer integrity.	[42]
Colloidal polydopamine beads support noble metal nanocatalysts for enhanced photothermal catalytic reactions.	Uncertainties about the stability of the catalyst system and potential environmental impacts of nanoparticles.	[43]
PDA coating enhances the biocompatibility and functionality of iron oxide nanoparticles.	Optimization of coating thickness and uniformity to maintain nanoparticle functionality.	[50]
Versatile applications in nanomedicine due to PDA’s adhesive properties and functionalization ease.	Long-term stability and biocompatibility of PDA under physiological conditions need more study.	[144]
Offers a new method for antimicrobial surface functionalization using the aza-Michael reaction.	Durability of antimicrobial properties and potential resistance development in bacteria.	[145]
Improved interfacial strength and hydrothermal aging resistance in composites.	Concerns about the long-term environmental impact and health safety of silica nanoparticles.	[146]
Enhanced mineralization and crystalline orientation in collagen matrices.	Potential variability in mineralization efficiency depending on environmental conditions.	[147]
Efficient enzyme bioconjugation for antimicrobial applications on implant surfaces.	Limited research on long-term stability and biocompatibility of plasma-deposited materials.	[148]
